# Cardiac-targeted delivery of nuclear receptor RORα via ultrasound targeted microbubble destruction optimizes the benefits of regular dose of melatonin on sepsis-induced cardiomyopathy

**DOI:** 10.1186/s40824-023-00377-8

**Published:** 2023-05-05

**Authors:** Shanjie Wang, Kegong Chen, Ye Wang, Zeng Wang, Zhaoying Li, JunChen Guo, Jianfeng Chen, Wenhua Liu, Xiaohui Guo, Guangcan Yan, Chenchen Liang, Huai Yu, Shaohong Fang, Bo Yu

**Affiliations:** 1 The Key Laboratory of Myocardial Ischemia, Chinese Ministry of Education, Heilongjiang Key Laboratory for Accurate Diagnosis and Treatment of Coronary Heart Disease, Department of Cardiology, Second Affiliated Hospital of Harbin Medical University, 246 Xuefu Road, Nangang District, Harbin, 150086 China; 2 Department of Thoracic Surgery, First Affiliated Hospital of Anhui Medical University, Hefei, 230022 China; 3 Laboratory Animal Center, Second Affiliated Hospital of Harbin Medical University, Harbin, 150086 China; 4 Department of Intensive Care Medicine, Second Affiliated Hospital of Harbin Medical University, Harbin, 150086 China; 5 Department of Pathology, First Affiliated Hospital of Anhui Medical University, Hefei, 230022 China; 6 Department of Epidemiology and Biostatistics, School of Public Health, Harbin Medical University, Harbin, 150086 China

**Keywords:** Melatonin, RORα, Ultrasound-targeted microbubble destruction, UTMD, Sepsis-induced cardiomyopathy, Oxidative stress, Mitochondria

## Abstract

**Background:**

Large-dose melatonin treatment in animal experiments was hardly translated into humans, which may explain the dilemma that the protective effects against myocardial injury in animal have been challenged by clinical trials. Ultrasound-targeted microbubble destruction (UTMD) has been considered a promising drug and gene delivery system to the target tissue. We aim to investigate whether cardiac gene delivery of melatonin receptor mediated by UTMD technology optimizes the efficacy of clinically equivalent dose of melatonin in sepsis-induced cardiomyopathy.

**Methods:**

Melatonin and cardiac melatonin receptors in patients and rat models with lipopolysaccharide (LPS)- or cecal ligation and puncture (CLP)-induced sepsis were assessed. Rats received UTMD-mediated cardiac delivery of RORα/cationic microbubbles (CMBs) at 1, 3 and 5 days before CLP surgery. Echocardiography, histopathology and oxylipin metabolomics were assessed at 16–20 h after inducing fatal sepsis.

**Results:**

We observed that patients with sepsis have lower serum melatonin than healthy controls, which was observed in the blood and hearts of Sprague–Dawley rat models with LPS- or CLP-induced sepsis. Notably, a mild dose (2.5 mg/kg) of intravenous melatonin did not substantially improve septic cardiomyopathy. We found decreased nuclear receptors RORα, not melatonin receptors MT1/2, under lethal sepsis that may weaken the potential benefits of a mild dose of melatonin treatment. In vivo, repeated UTMD-mediated cardiac delivery of RORα/CMBs exhibited favorable biosafety, efficiency and specificity, significantly strengthening the effects of a safe dose of melatonin on heart dysfunction and myocardial injury in septic rats. The cardiac delivery of RORα by UTMD technology and melatonin treatment improved mitochondrial dysfunction and oxylipin profiles, although there was no significant influence on systemic inflammation.

**Conclusions:**

These findings provide new insights to explain the suboptimal effect of melatonin use in clinic and potential solutions to overcome the challenges. UTMD technology may be a promisingly interdisciplinary pattern against sepsis-induced cardiomyopathy.

**Supplementary Information:**

The online version contains supplementary material available at 10.1186/s40824-023-00377-8.

## Introduction

Sepsis is the leading cause of mortality in intensive care units, accounting for one-fourth of cases [1]. Concomitant heart dysfunction increases the risk of death in patients with severe sepsis [2]. Although a perceptible decrease in cardiac function has been well-characterized in patients with sepsis, there is no consensus or guidelines on the management of sepsis-induced cardiomyopathy to date [2, 3].

Melatonin is an ancient and highly conservative hormone across species [4]. The biological effects mainly contribute to receptor-dependent pathway [5, 6], while the direct scavenging oxyradical contributed only a small portion [7]. MTNR1A/MT1 and MTNR1B/MT2 were the well-known melatonin receptors; although the nuclear receptors ROR family as melatonin receptors remains controversial, melatonin regulated the transcriptional activity of ROR at least via indirect action [8]. Other and our previous animal studies [6, 9, 10] have confirmed the remarkable effects of melatonin treatment, but clinical trials observed insignificant or limited benefits [11–13]. Decreased receptors in pathological conditions might weaken the effects of melatonin depending on the receptor-related pathway [6, 14]. Notably, the equivalent dosage of melatonin used in animal studies was much higher than in clinical trials [9, 15, 16]. Although a large dose of melatonin supplementation neutralizing oxygen free radicals may partially compensate for insufficient melatonin receptors, high-dose use may be unlikely to translate clinically due to potential safety concerns [17–19]. Therefore, we hypothesized that amplifying the receptor-dependent pathway might optimize the benefits of mild-dose of melatonin supplementation, which may partly explain the suboptimal results of melatonin therapy in clinic.

Ultrasound-targeted microbubble destruction (UTMD) technology is a new drug and gene delivery system to the target tissue, which has proven to be highly efficient and safe in delivering genes to target organs [20]. In contrast, UTMD avoided the ethical and biosafety issues of viral transfection [21–23]. Clinical trials demonstrated ultrasound microbubbles’ favorable safety for thrombolytic therapy and adjuvant chemotherapy [21, 24]. Low-frequency ultrasound used in the UTMD technology had the advantageous features of strong penetration, reduced decay, and focus on deep local tissues [25]. After intravenous injection, these microbubbles enter the target tissue with the blood flow and undergo oscillation and collapse under ultrasound stimulation, resulting in a local cavitation effect [20]. This process generates sonoporation and shear stress on cell membranes to enhance the permeability, increasing penetration and uptake of plasmids.

In this study, we demonstrated that UTMD-mediated cardiac delivery of melatonin-related receptor genes could optimize the cardioprotective effects of a clinically safe dose of melatonin on sepsis-induced cardiomyopathy. Although we observed decreased serum melatonin levels in sepsis patients, a clinical dose of melatonin administration in animal models produced weak benefits. We further evaluated classic and non-classic melatonin receptors and found that the mRNA and protein expression of nuclear receptor RORα, rather than MT1/2, was significantly reduced in the sepsis model. To overcome this issue, we constructed a UTMD system to deliver and overexpress RORα in the heart. Traditional microbubbles have limited loading capacities, whereas cationic microbubbles (CMBs) are better suited for carrying plasmids with polyanions through electrostatic interactions [26]. Plasmids containing the RORα gene were loaded into the CMBs, and the targeted delivery of the RORα gene was achieved through UTMD technology. We found that nuclear receptor RORα promoted the effect of regular dose melatonin on the antioxidant system. The synergistic therapy alleviated lipid peroxidation and mitochondrial damage in heart tissues, improved cardiac dysfunction and survival time in rats with severe sepsis. Therefore, the UTMD gene delivery system improved the responsiveness of conventional dose melatonin treatment by reshaping the melatonin receptor pathway. Our findings may aid in understanding and resolving the current melatonin therapy dilemma in the clinic.

## Methods

### Clinical participants

This study included 16 patients diagnosed with severe sepsis with age ≥ 20 years and eligible for blood sample collection without renal replacement therapy in the intensive care unit, Second Affiliated Hospital of Harbin Medical University, between July and December 2021. Age- and sex-matched volunteers without infectious disease or cardiopathy in the medical examination center were selected as healthy controls. Blood samples were collected between 7:00 AM and 9:00 AM and retained in the Biobank of the Key Laboratory of Myocardial Ischemia, Ministry of Education. The protocols were approved by the ethics review board of Harbin Medical University. All participants provided informed consent.

### Animal models and treatment

Sprague–Dawley rats (weighing 220–250 g) were fed in the Animal Centre of the Second Hospital of Harbin Medical University at about 22 °C and a 12 h light-dark cycle with free access to fodder and water. All animal experiment protocols conformed to the Guidelines for the Care and Use of Laboratory Animals and were approved by the Ethics Committee of the Harbin Medical University of Animal Care and Use. At each stage, animals were randomly divided into control and treatment groups. Specific groups and sample sizes are described in figure legends.

The cecal ligation and puncture (CLP) model was constructed to induce lethal sepsis [16]. Rats were anesthetized with 2–3% isoflurane and immobilized. The cecum was exposed by a 1.5 cm abdominal midline incision, ligated below the ileocecal valve, and punctured with an 18-gauge needle. Rats in the control group underwent the same procedures without CLP. After the surgery, all rats received 0.9% saline solution to avoid hypovolemia (40 mL/kg). The lipopolysaccharide (LPS)-induced sepsis model was also established to confirm the robustness of the association between sepsis and melatonin and melatonin receptors. Rats were injected intraperitoneally with LPS (25 mg/kg of body weight, Sigma-Aldrich, St. Louis, USA, L2880) to induce severe endotoxemia.

### Plasmid/CMBs construction and UTMD technology

**Microbubble generation**: Cationic microbubbles (CMBs) were generated by thin-film hydration-sonic vibration as previously described [23]. Several materials 1,2-dipalmitoyl-sn-glycero-3-phosphocholine (DPPC, Avanti, 850355P), 1,2-distearoyl-sn-glycero-3-phosphoethanolamine-N-[methoxy(polyethylene glycol)-2000] (DSPE-PEG2000, Avanti, 880127P), 3-[N- (N,N-dimethyl-aminoethane)-carbamoyl] cholesterol (DC-Chol, Avanti, 700001P) were mixed according to the mass ratio of 5:2:0.5, and dissolved in 5 mL of chloroform in a flask. The organic solution was removed on a rotary evaporator at 50 °C, and a thin and homogeneous lipid film was obtained. 0.5 mL of glycerol solution (glycerol:PBS = 1:9, v/v, Sigma-Aldrich, St. Louis, USA, G5516) was added into the flask at 42 °C for 30 min and transferred to a 1.5 mL tube. The bottle was wrapped with a sealing film and sterilized by ultraviolet light for 1 h. Octafluoropropane gas (C_3_F_8_) is commonly used as core gas of microbubbles for contrast echocardiography [27]. C_3_F_8_ was bubbled through the solution and vibrated for one minute to obtain the gas-filled CMBs. The CMBs were diluted with PBS to 1 × 10^9^ MBs/mL. CMB solution was sterilized by ^60^Co-γ radiation and stored at 4 °C until use. The size distribution and surface potential of CMBs were determined using the Zetasizer Nano ZS system (Malvern, Worcestershire, UK).

**Plasmid/CMBs construction**: Several doses (10–80 µg in 50 µL) of plasmids containing GFP or RORα gene were mixed and incubated with 50 µL CMB suspension (5 × 10^8^ MBs) for 15 min. The mixtures were centrifuged at 400 *g* for 5 min and separated into two layers: the upper milky white containing plasmid banded to CMBs and the lower pellucid phase containing free plasmids. The lower phase was centrifuged at 10,000 *g* for 5 min using a 0.45 μm filter to harvest the resuspended, unconjugated plasmid to determine the DNA concentration. The percentage of plasmid DNA banded to CMBs was defined as the (DNA_total_ - DNA_free_)/DNA_total_ × 100%; the payload mass of plasmid in CMBs was defined as the (DNA_total_ - DNA_free_)/CMB number.

**UTMD delivery**: After adaptive feeding for seven days, rats were anesthetized with 2–3% isoflurane and injected intravenously with prepared plasmid/CMBs mixtures via the tail vein (1.0-1.2 mL/hour), as previously described [26]. The ultrasound beam was transmitted using the M3S transducer (S5–1 probe, GE Healthcare, Milwaukee, WI) with a frequency range 1–5 MHz. The reported resonant frequency of microbubbles was 1–3 MHz [28]. The pre-specific ultrasound parameters were set as the second harmonic mode (transmitting 1.6 MHz and receiving 3.2 MHz) with an electrocardiograph (ECG)-mediated trigger for ultrasound burst at each fourth end-systole for 20 min. The transducer was covered with a gel interface to keep the focus at the myocardium level (depth 3 cm, mechanical index (MI, Low): 0.30, MI (Flash): 1.31, and output power: -16db). UTMD-mediated RORα delivery was repeated three times at one-day intervals [22].

### Cell culture and treatment

H9C2 cells were cultured with high-glucose DMEM containing 10% (v/v) fetal bovine serum (ScienCell, USA, #0500) in a humidified incubator with 5% CO_2_ and 37 °C. When the cell density reached 60%, H9C2 cells were transduced with plasmid using Lipo3000 (Invitrogen, USA, L3000008) according to the manufacturer’s protocols. Twenty-four hours later, cells were further treated with or without LPS (0.25-4 µg/mL) to imitate the sepsis challenge. Meanwhile, cells were cultured with or without melatonin (50 µmol/L) for 24 h and then harvested for analysis.

### Echocardiography

Echocardiography (S12–4 probe, EPIQ 5; Philips) was used to evaluate cardiac function 16 h after CLP surgery. As described in our previous study, rats were anesthetized with 2–3% isoflurane and immobilized. The left ventricular ejection fraction (LVEF) and left ventricular fraction shortening (LVFS) were measured during at least four consecutive cardiac cycles.

### Apoptosis assessment

Terminal deoxynucleotidyl transferase-mediated dUTP nick end labeling (TUNEL, Elabscience Biotechnology Co., Ltd, China, E-CK-A321) staining was used to evaluate the apoptosis ratio of H9C2 cells or heart section as described in our previous study [29]. Apoptosis index was defined as TUNEL-positive nucleus/total nucleus in five random fields per sample.

### Reactive oxygen species assessment

DHE staining was used to detect reactive oxygen species (ROS) generation in the fresh heart tissue. The frozen sections were pretreated with PBS for 10 min and then incubated with DHE staining solution (10 µmol/L, Beyotime, China, S0063) at 37 °C in the dark cassette. After the incubation for 30 min, heart sections were washed with PBS three times and remained wet until observation using a fluorescence microscope (Leika, Germany).

### DPPH-radical scavenging activity

Antioxidant properties was determined using the 2,2-diphenyl-1-picrylhydrazyl (DPPH) free radical scavenging activities (Solarbio, China, BC4750) as previously reported [30]. DPPH solution was treated with melatonin, low intensity ultrasound, and/or CMBs. Sample without DPPH were set as the blank control. The absorbance was measured at 515 nm using Spectrophotometer. Antioxidant activity was calculated according to the manufacturers’ instructions and expressed in percentage.

### Mitochondrial membrane potential

The mitochondrial membrane potential was assessed using a JC-1 staining assay kit (Beyotime, China, C2005). The fluorescent dye JC-1 accumulates in the mitochondrial matrix in two forms depending on mitochondrial membrane potential: JC-1 aggregates (red) in healthy mitochondria and JC-1 monomers (green) in depolarized mitochondria. The frozen sections were pretreated with PBS for 10 min and then stained with a JC-1 probe (10 µg/mL) at 37 °C for 30 min in the dark cassette [31]. After washings with PBS three times, the sections were observed using a fluorescence microscope (Leika, Germany). Five random images were captured per sample to examine the fluorescence intensity of the J-monomers and the J-aggregates.

### Pathological analysis

Heart tissues were fixed with 4% paraformaldehyde at room temperature for 72 h, paraffin-embedded, and sliced into sections with 4 μm thickness. The sections were stained with hematoxylin and eosin (Right tech, China) to observe the morphology of cardiac fibrosis. Paraffin sections were deparaffinized, blocked, and incubated with rabbit anti-RORα (1:200, Affinity Bioscience, China, #DF3161) or anti-4 hydroxynonenal (4-HNE,1:500; R&D, MAB3249-SP) overnight at 4 °C under humidified conditions. Tissues were further incubated with HRP-labeled secondary antibody for 1 h.

### Cell viability assay

Cell viability was determined by the MTT Cell Proliferation Assay Kit (Beyotime Biotechnology, Shanghai, China, C0009). H9C2 cells (5 × 10^3^ cells per well) were seeded in 96-well plates and incubated with MTT reagents after treatment.

Lactate dehydrogenase (LDH) activity of the serum or supernatant of the cellular medium was estimated using a commercially available assay kit (Jiancheng, China). In brief, 50 µL of diluted serum was added into 96-well plates and added with 50 µL/well of the reaction mix. The absorbance was measured at 450 nm using Spectrophotometer. LDH activity was calculated according to the manufacturers’ instructions.

### Enzyme-linked immunosorbent assay (ELISA) kits

The serum levels of endotoxin were measured using a Limulus test kit (Xiamen Bioendo Technology Co., Ltd. China, EC64405) according to the manufacturer’s protocols. Serum CK-MB, IL-1β, TNF-α, and melatonin were measured using a commercial ELISA kit according to the instructions (Elabscience Biotechnology Co., Ltd, China, China, E-EL-R1327c; Multisciences, China, EK301B; Multisciences, China, EK382; Multisciences, China, 70-E0194; Elabscience Biotechnology Co., Ltd, China, China, E-EL-R0031c).

### RT-qPCR

RNAs were harvested using TRIzol reagent (HaiGene, China, B0201) and reverse-transcribed to cDNA using the RT Easy II First Strand cDNA Synthesis Kit (Roche, Switzerland, 04379012001). The target genes were amplified using a Real-Time PCR Easy (SYBR Green I, MCE, China, HY-K0501). Gene expression was normalized to the β-actin level and calculated using the ^−ΔΔ^CT method. The primers are presented in Table S1.

### Western blotting

Total protein was extracted from H9C2 cells or heart tissues using RIPA buffer containing protease and phosphatase inhibitors. Protein concentration was measured and adjusted according to the bicinchoninic acid protein assay. Briefly, 30 µg proteins per sample were loaded into 10–12% SDS–PAGE gels, separated, and transferred to 0.22 μm PVDF membranes. The band was blocked with 5% BSA for 1 h, incubated with primary antibodies overnight at 4 °C (Table S2), and further incubated with HRP-conjugated secondary antibodies (1: 8000) for 1 h at room temperature. Immunoreactivity was detected using a chemiluminescence ECL kit and the automatic chemiluminescence imaging system (Tanon 5200, Guangzhou, China).

### Oxylipin assessment

The experimental protocols were validated in previous studies [32]. Heart tissues were stored at − 80 °C, thawed in a centrifuge tube, and ground away from light. Methanol (0.2 mL) containing internal standard was added to the 20 mg heart sample to precipitate proteins. Butylated hydroxytoluene (0.005%) were added into solution to avoid oxidative reaction during extraction. The eicosanoids in supernatants were extracted using Poly-Sery MAX SPE columns (ANPEL). The extraction was analyzed using a LC-ESI-MS/MS system (UPLC, ExionLC AD; MS, QTRAP®6500 + System; Column, Waters ACQUITY UPLC HSS T3 C18 100 mm × 2.1 mm, i.d. 1.8 μm). The binary gradient consisted of (A) 0.04% water/acetic acid and (B) 0.04% acetonitrile/acetic acid for 10 min. Metabolic features of oxylipins were analyzed using scheduled multiple reaction monitoring (MRM). The contents of oxylipins in heart tissues were quantified according to the calibration curve for each eicosanoid (Table S3).

### Statistical analyses

All quantitative data were presented as means ± standard deviations (SD) unless otherwise noted. R (4.2 version) and GraphPad Prism (6.0 version) were used for statistical analysis and visualization. Comparisons between two groups were performed using Student’s t-test or Mann-Whitney U test according to the data distribution. Principal component analysis (PCA) and partial least squares-discriminant analysis (PLS-DA) were used to assess the different metabolites in oxylipin profiles. The difference was considered statistically significant if the p-value was less than 0.05.

## Results

### Melatonin and related receptors in the sepsis heart: decreased RORα expression

**Fig. 1:**
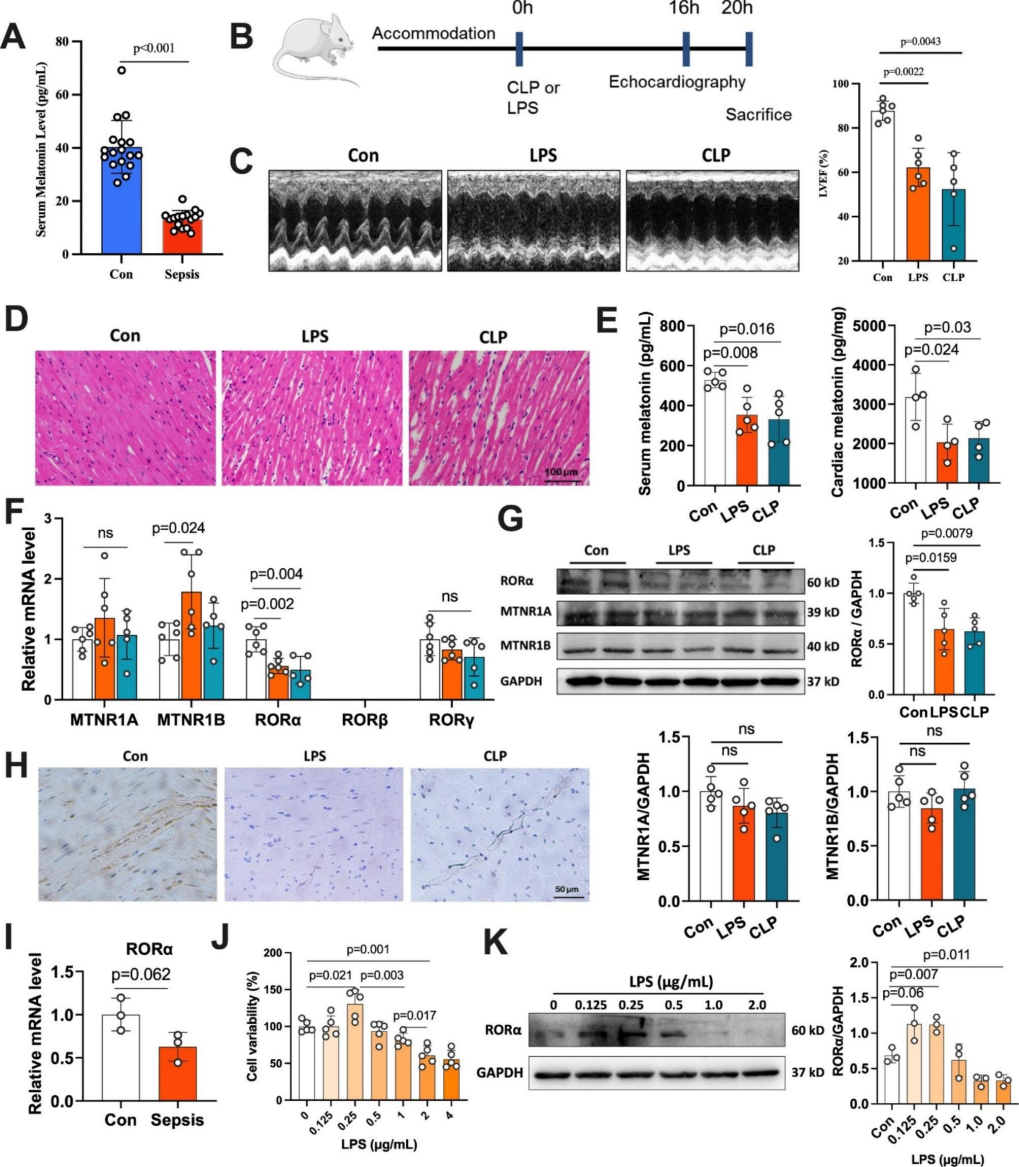
Melatonin and related receptors in humans and rats with sepsis. (A) Serum levels of melatonin in patients with sepsis and age-/sex-matched health controls (n = 16). (B) Experimental scheme and the timeline for the sepsis model. (C) Representative images of M-mode echocardiography of rats 16 h after LPS treatment or CLP surgery and the left ventricle ejection fraction in control and sepsis-induced rats (n = 6). (D) Representative hematoxylin and eosin (H&E) staining images of the heart section (scale bar of 100 μm). (E) Melatonin levels in serum (n = 5) and cardiac tissues (n = 4) among rats with sepsis and control. (F) Relative mRNA levels of melatonin receptors in heart tissue (n = 5–6). (G) Western blots and quantifying melatonin receptors MTNR1A, MTNR1B, and RORα (n = 4). (H) Immunohistochemical staining of RORα in the sepsis heart (scale bar of 50 μm). (I) Relative mRNA levels of RORα in HL-1 cells with LPS treatment (n = 3, GSE56584). (J) Cellular viability of H9C2 myoblasts in response to different doses of LPS (n = 5). (K) Western blots and quantification of RORα expression in H9C2 cells treated with low and high doses of LPS (n = 4)

We measured serum melatonin in patients with severe sepsis and age-/sex-matched health controls (Table S4). According to ELISA assay, serum melatonin in septic patients was less than one-third of that in healthy adults (13.3 versus 40.5 pg/mL, Fig. 1A). We further constructed rats with sepsis using intraperitoneal (i.p.) injection of LPS (25 mg/kg) or CLP surgery (Fig. 1B), which were identified by increased serum endotoxin levels and decreased left ventricular systolic function (Figure S1 and 1 C). Hematoxylin and eosin (H&E) staining showed that myocardial fibers exhibited swelling, disorganization, and fracture 20 h post LPS or CLP challenge (Fig. 1D). Consistent with the results in humans, septic rats had lower melatonin levels in serum and heart than the physiological level (Fig. 1E).

We found a decreased mRNA level in cardiac nuclear RORα but not RORβ/γ or MT1/MT2 in both sepsis models (Fig. 1F), which was further confirmed by western blotting and immunohistochemical assays (Fig. 1G-H and S2). We also screened the five receptors from the GEO database under sepsis condition. Since acute inflammatory cells infiltration was associated with sepsis-induced injury, we retrieved the expression of melatonin-related receptors in inflammatory cells [1].The expressions of the five receptors were unchanged after LPS treatment in neutrophils (acute inflammatory cells) (Figure S3A-C). However, only RORα moderately decreased in cardiomyocytes under sepsis stress (Fig. 1I and Figure S3D).

RORα expression was reversely correlated with the degree of sepsis severity in the mRNA microarray (Figure S3E). Interestingly, when H9C2 myoblasts were treated with different concentrations of LPS, low-dose LPS (0.5 µg/mL) improved cellular viability, but high-dose exposures to LPS (≥ 2 µg/mL) significantly inhibited cellular viability and RORα expression in vitro (Fig. 1J-K). These data suggest that endotoxemia may stimulate dose-dependent changes in the cardiac RORα expression, initially activating at low-dose and declining at fatal doses.

### RORα overexpression improves the protective effect of melatonin on LPS-treated cardiomyocytes in vitro

**Fig. 2:**
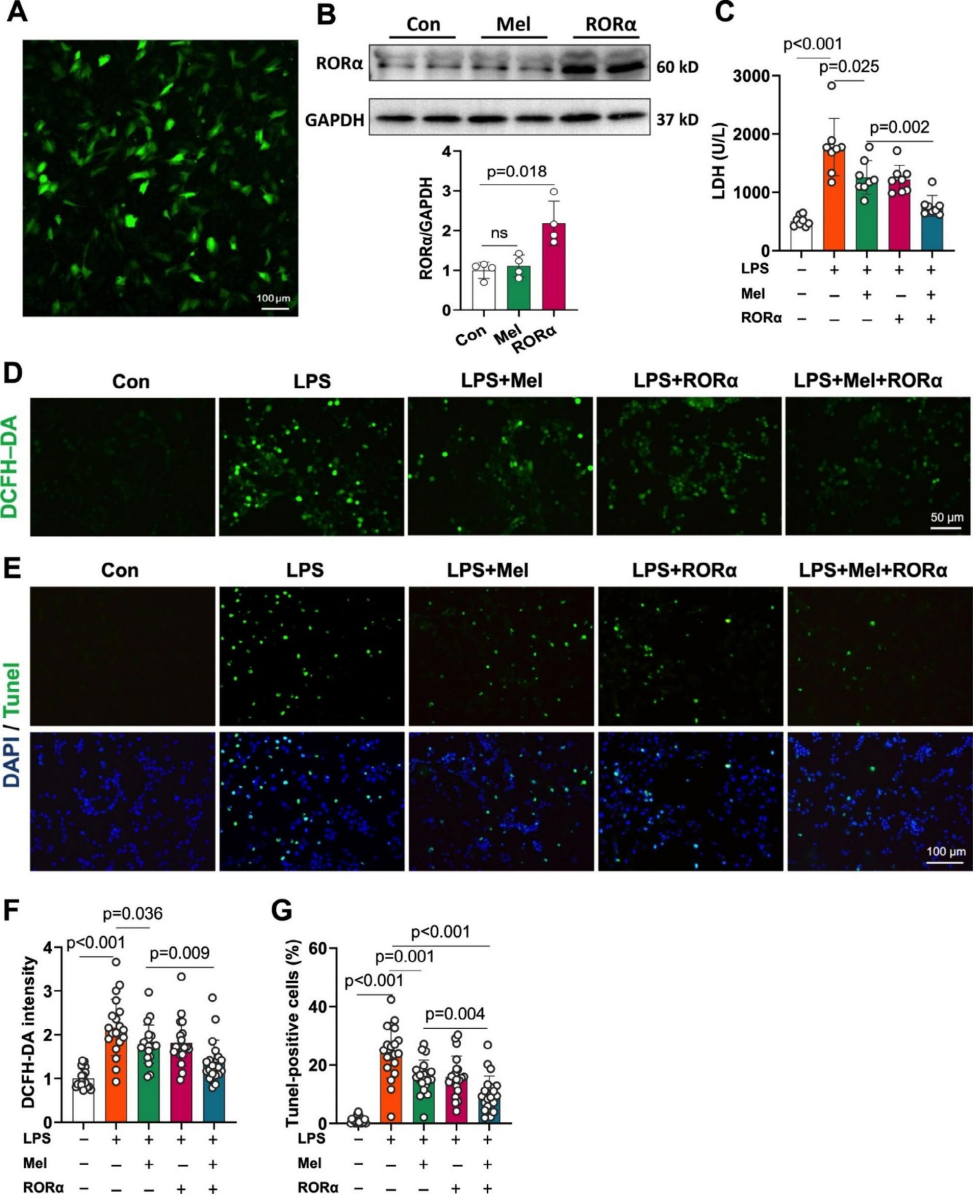
RORα overexpression improved the effect of melatonin treatment on LPS-induced cardiomyocyte injury *in vitro.* (A) H9C2 cells treated with a plasmid containing the GFP gene 48 h later showing a transfection rate of more than 80% (scale bar of 100 μm). (B) Western blots and quantification of RORα expression in H9C2 cells transfected with vehicle or RORα plasmid for 48 h or treated with melatonin for 24 h (n = 4). (C) The effect of LDH leak from RORα-overexpressed H9C2 cells treated by LPS and melatonin (n = 8). (D) DCFH-DA staining was performed to assess ROS generation in RORα-overexpressed H9C2 cells treated by LPS and melatonin (n = 4, ~ 20 views per group, scale bar of 50 μm). (E) TUNEL staining to assess apoptosis index in RORα-overexpressed H9C2 cells treated by LPS and melatonin (n = 4, ~ 20 views per group, scale bar of 100 μm)

High-dose melatonin activates pro-survival pathways against sepsis-related myocardial injury [16]. However, it is unclear if melatonin-related receptor insufficiency impairs melatonin’s pharmacological effect. We confirmed that the RORα gene but not melatonin increased RORα expression (increased by 150%) in H9C2 cells, suggesting that melatonin use might not rescue the decrease in RORα under sepsis (Fig. 2A-B). As expected, 2 µg/mL LPS increased LDH leak from H9C2 cells in vitro, which was moderately reversed by melatonin alone (Fig. 2C). However, the combination of melatonin with RORα overexpression minimized LDH release by 60% in LPS-treated H9C2 cells (Fig. 2C). Oxidative stress and apoptosis are the prominent hallmarks of LPS-induced myocardial injury [16, 33]. RORα overexpression further amplified the effect of melatonin to reduce LPS-induced ROS generation and apoptosis (Fig. 2D-G). These results suggested that RORα overexpression may optimize melatonin therapy on cardiomyocytes in response to endotoxemia, which warrants further validation in vivo.

### Preparation and characterization of RORα plasmids/CMBs

**Fig. 3:**
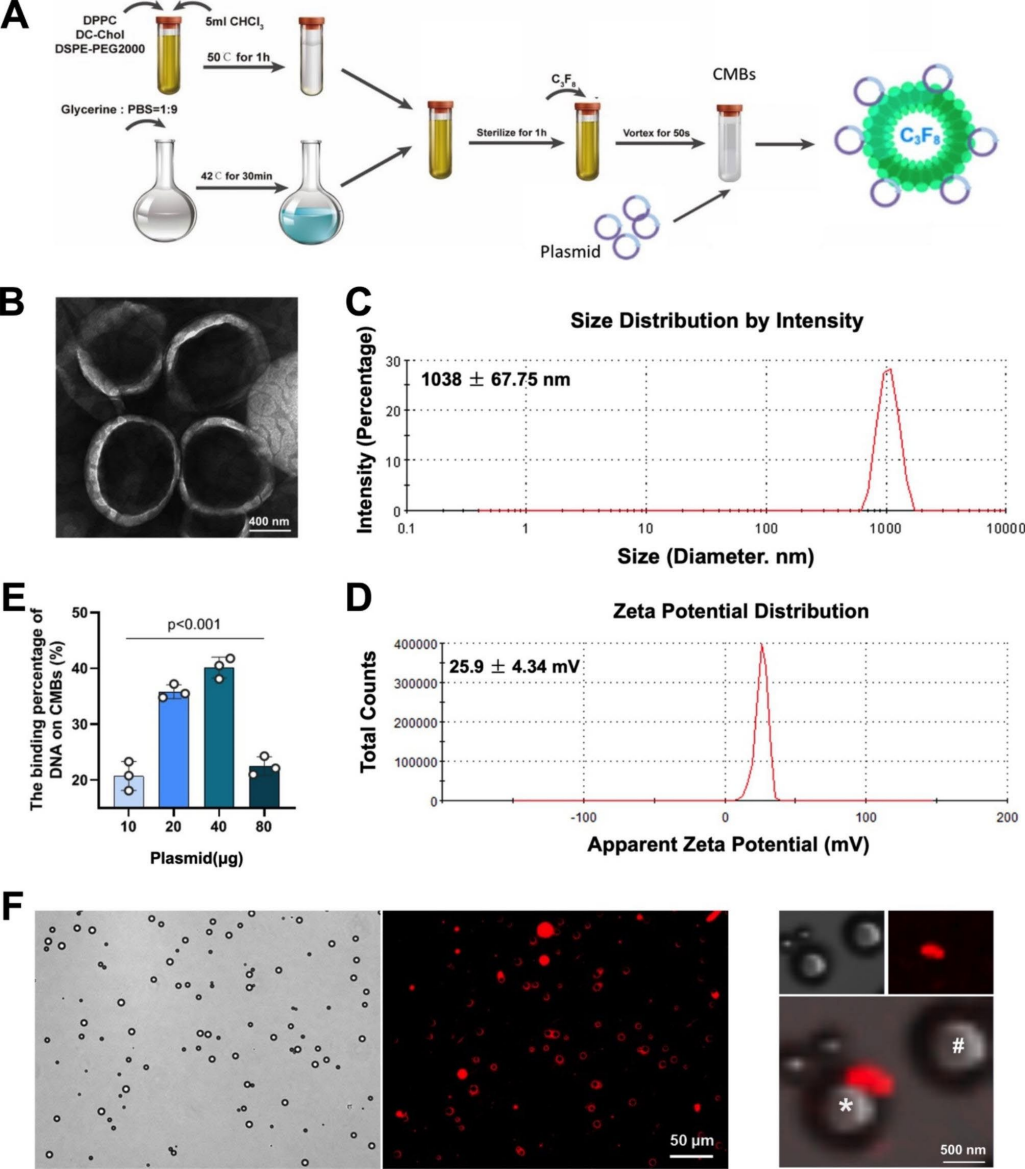
Preparation and assessment of plasmid/CMBs. (A) Schematic diagram of the preparation of CMB particles. (B) Representative images of CMBs under the transmission electron microscope (scale bar of 400 nm). (C) Size distribution of the CMBs. (D) Potential distribution of the CMBs. (E) The binding percentage of different doses of DNA on CMBs (n = 3). (F) Representative images of plasmid/CMBs. Plasmid labeled by PI stain (red) was merged with the outline of CMB (bright) (scale bar of 50 μm). A local image of CMB with or without plasmid was indicated by the white asterisk (*) and pound sign (#) (scale bar of 500 nm)

We constructed CMBs to carry plasmids containing RORα according to the process shown in Fig. 3A. Transmission electron microscopy showed the spherical shape of CMBs with a particle diameter in the range of 0.5–1.5 μm (Fig. 3B). According to the dynamic light scattering, the average size of synthesized CMBs was 1038 ± 67.75 nm (Fig. 3C), and the zeta potential was + 25.9 ± 4.34 mV (Fig. 3D). The binding capacity to CMBs was estimated when 10–80 µg plasmids were incubated with 5 × 10^8^/CMBs, in which the maximum binding capacity of CBMs was observed when adding 40 µg DNA (Fig. 3E). After incubation with plasmid and centrifugation, the particle size of RORα/CMBs was 912 ± 71.3 nm, with an average zeta potential of 16.2 ± 3.15 mV (Figure S4). Fluorescence images of stabilized RORα/CMBs showed the merge of RORα plasmid labeled by Propidium Iodide staining and lipid membrane of CMBs (Fig. 3F). The CMBs we prepared were packaged and stored by freeze-drying, and the plasmid/CMBs complex used for UTMD was prepared when needed. The synthesized plasmid/CMBs complex has good stability for at least 4 h under room temperature (Figure S5).

### Efficiency of cardiac delivery of RORα plasmid via UTMD

**Fig. 4:**
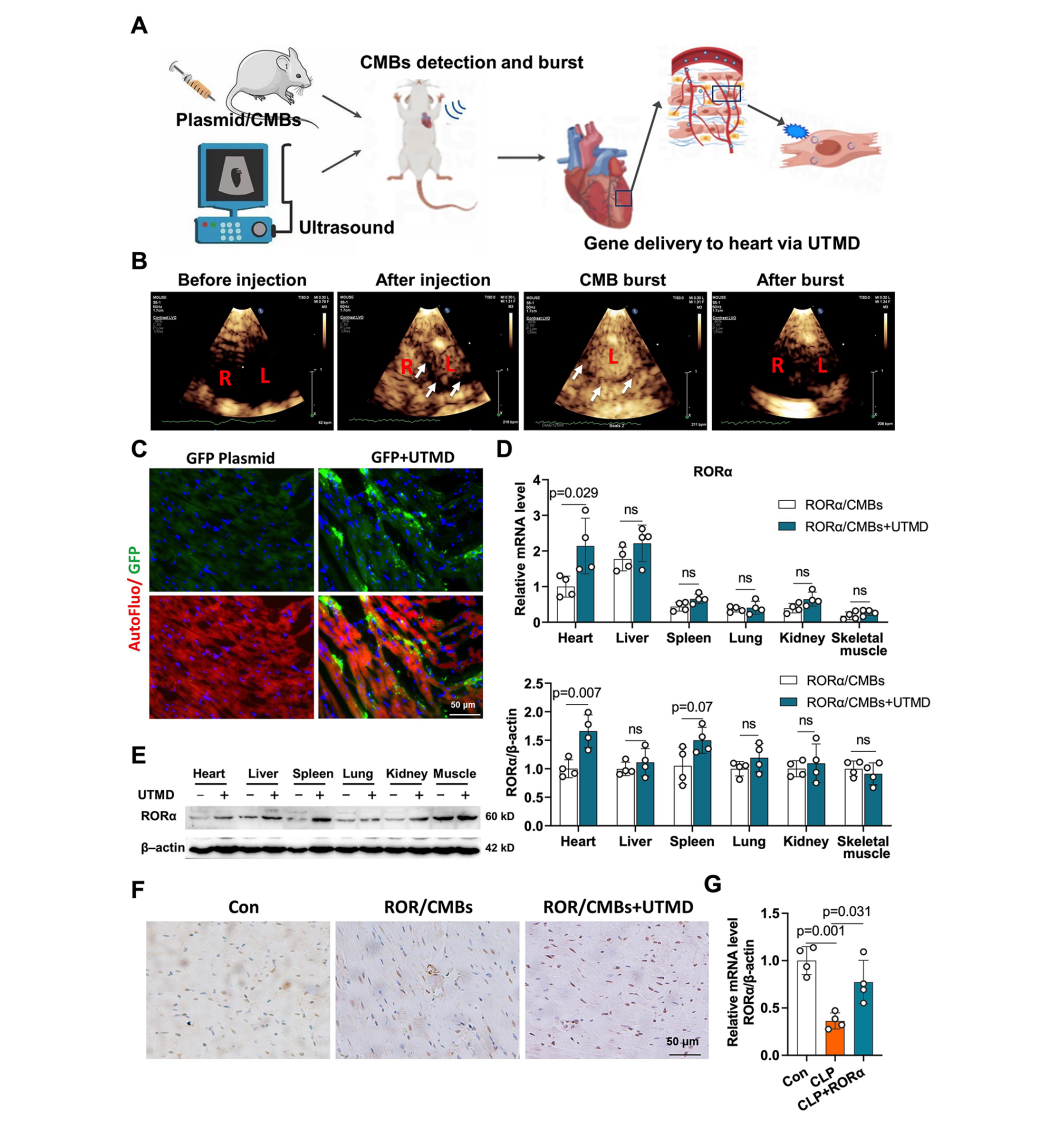
Efficiency and specificity of cardiac uptake of RORα plasmid/CMBs *in vivo*. (A) Schematic diagram of UTMD-mediated RORα delivery to the heart. (B) Representative ultrasound contrast images of CMBs in the heart were obtained before and after plasmid/CMBs injection, as well as after ultrasound-targeted microbubble blast. The second harmonic mode (transmitting at 1.6 MHz and receiving at 3.2 MHz) with an electrocardiograph (ECG)-mediated trigger was used to induce CMBs burst. Following CMBs injection, microbubbles with high-echo intensity filled the left ventricle (L), right ventricle (R), and the ventricular wall tissue (arrows). After complete CMBs blasting, only a small amount of microbubbles remained in the heart cavity and showed a low echo shadow. (C) Fluorescence images of successful GFP plasmid transfection dependent on UTMD technique, DAPI for nucleus; green for GFP (scale bar of 50 μm). (D) Relative mRNA levels of RORα expression in the heart and other organs (n = 4). (E) Western blots and quantification of RORα expression in multiple organs (n = 4). (F) Immunohistochemical images of RORα expression in cardia tissue after UTMD treatment (scale bar of 50 μm). (G) RT-qPCR demonstrated that UTMD mediated the overexpression of cardiac RORα in rats with severe sepsis (n = 3)

We first assessed the safety of UTMD operation with prespecified parameter. Although the cavitation may cause cellular stress, the low intensity focused ultrasound we used does not significantly cause cellular injury and ROS generation in vivo (Figure S6). According to DPPH assay, UTMD and CMBs did not significantly affect the antioxidant activity of the melatonin in vitro (Figure S7). We assessed the delivery efficiency of plasmids/CMBs in heart tissue mediated by UTMD in vivo (Fig. 4A). Echocardiography showed the distribution of CMBs in myocardial tissues and ECG-triggered burst of CMBs to release plasmid (Fig. 4B and Video S1-4). GFP protein was successfully detected in heart after three days (Fig. 4C). After three times of RORα transfections, the mRNA and protein levels of cardiac RORα increased by 70-110% (Fig. 4D-F). However, RORα expression was not significantly different in liver, spleen, lung, skeletal muscle, and kidney after UTMD, although mRNA levels of RORα moderately increased in liver and spleen (Fig. 4D). RORα overexpression was also achieved in septic heart (Fig. 4G). Thus, UTMD-mediated target delivery of gene may be a promising strategy to rescue RORα expression in the sepsis heart.

### The combination of RORα delivery and melatonin administration improves myocardial injury and survival in rats with sepsis beyond melatonin alone

**Fig. 5:**
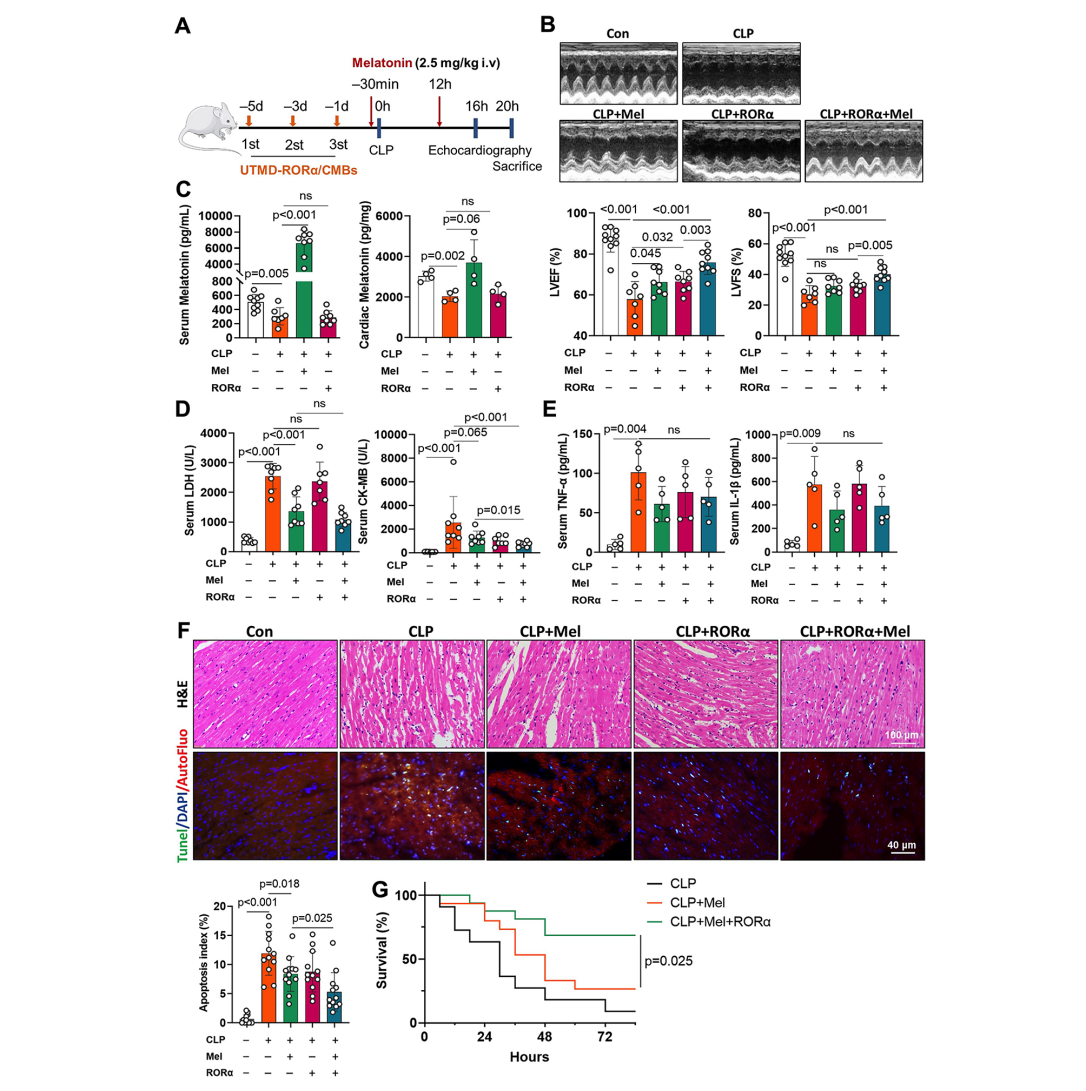
The combination of RORα/CMBs and melatonin treatment optimized the benefits to improve cardiac injury beyond melatonin alone in rats with fatal sepsis. (A) Experimental scheme and timeline for sepsis model construction and interventions. UTMD-mediated RORα delivery (40 µg/kg) was repeated at one-day intervals 1 day, 3 days, and 5 days before CLP surgery. Intravenous melatonin 2.5 mg/kg was given 30 min before surgery and 12 h after the surgery. Heart tissue and blood were taken 20 h after CLP surgery. (B) Representative images of echocardiography at 16 h after CLP surgery. The left ventricle ejection fraction (LVEF) and left ventricular fraction shortening (LVFS) were calculated (n = 7–10). (C) Melatonin levels in serum (n = 7–8) and cardiac tissues (n = 4) at 20 h after CLP surgery. (D) Serum LDH and CK-MB levels, markers of cellular injury, were evaluated using ELISA kits 20 h after CLP surgery (n = 7–8). (E) Serum levels of TNF-α and IL-1β were elevated to reflect systemic inflammation (n = 5). (F) Representative hematoxylin and eosin (H&E, scale bar of 100 μm) and TUNEL staining images of heart sections (n = 3, ~ 12 views per group, scale bar of 50 μm). (G) Rats with sepsis were observed for 3.5 days after CLP surgery. The survival rates were analyzed using the Kaplan-Meier curve and log-rank test (n = 11–16 per group)

We assessed cardiac dysfunction 16 h after CLP surgery (Fig. 5A). As expected, intravenous melatonin administration at a dose of 2.5 mg/kg/12 h weakly improved LVEF and LVFS in rats with sepsis, whereas UTMD-mediated cardiac RORα overexpression further enhanced the protective effect of melatonin on heart function (Fig. 5B). Eight hours after the second melatonin administration, melatonin levels in serum and heart tissue remained higher than CLP group (Fig. 5C). Melatonin treatment moderately decreased serum CK-MB and LDH in sepsis rats, while RORα delivery further limited CK-MB not LDH leak (Fig. 5D). However, the cardiac benefits of a clinical safe dose of melatonin and cardiac RORα delivery were independent of inflammation level because TNFα and IL-1β were not significantly reduced by melatonin and RORα delivery (Fig. 5E).

Moreover, the broken and swollen myocardial fibers in fatal sepsis were largely lessened by melatonin and RORα delivery beyond melatonin monotherapy (Fig. 5F). Consistently, the combination therapy achieved the maximum benefits in limiting sepsis-induced myocardial apoptosis (Fig. 5F). During 4 days’ follow-up, rats receiving RORα/CMBs and melatonin (2.5 mg per 12 h) were more resistant to CLP-induced mortality risk (Fig. 5G). Thus, UTMD-mediated nuclear receptor RORα delivery enhanced melatonin-mediated cardiac protection against sepsis-induced cardiomyopathy.

### Nuclear receptor RORα improving antioxidant effects of melatonin against sepsis-induced cardiomyopathy

**Fig. 6:**
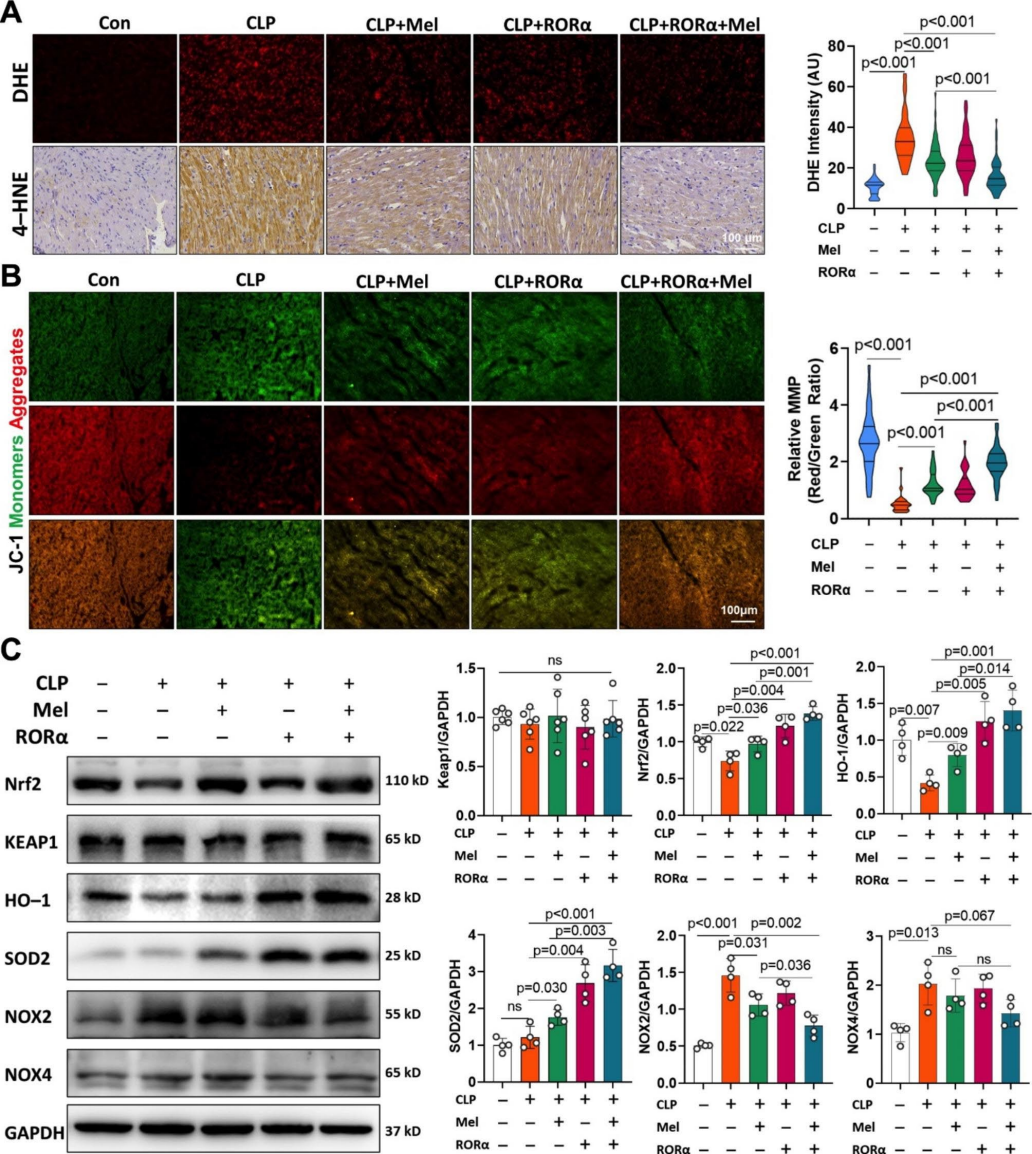
Nuclear receptor RORα enhanced the antioxidant effect of melatonin in sepsis-induced cardiomyopathy. (A) DHE and 4-HNE immunohistochemical staining in the heart Sect. 20 h after CLP surgery with or without RORα delivery and melatonin supplementation (scale bar of 100 μm). DHE intensity was quantified to reflect myocardial ROS levels (n = 7–10, ~ 40–59 views per group). (B) JC-1 staining of heart section (scale bar of 100 μm). The ratio of JC-1 aggregates (red) in healthy mitochondria and JC-1 monomers (green) in depolarized mitochondria was used to assess mitochondrial membrane potential (n = 5–7, ~ 22–34 views per group). (C) Western blots and quantification of Keap1, Nrf2, HO-1, SOD2, NOX2, and NOX4 expression in the sepsis heart with or without RORα delivery and melatonin supplementation (n = 4)

We investigated whether the antioxidant effect of a clinically equivalent dose of melatonin was dependent on RORα. According to in situ DHE staining and immunohistochemical analysis of 4-HNE, oxidative stress was moderately attenuated by melatonin supplementation, while the cardiac delivery of RORα further advanced the antioxidative effect of melatonin in septic heart (Fig. 6A and S8). The mitochondrial transmembrane potential was the primary source of ROS products (Fig. 6B). Myocardial tissues in CLP rats had a significant loss of mitochondrial membrane potential, which was improved by RORα delivery and a moderate dose of melatonin (Fig. 6B). Fatal sepsis significantly decreased antioxidant signals, such as Nrf2, HO-1, and SOD2/MnSOD, but increased NOX2/4 expression; those changes were improved considerably by melatonin and RORα delivery, not melatonin alone (Fig. 6C).

**Fig. 7:**
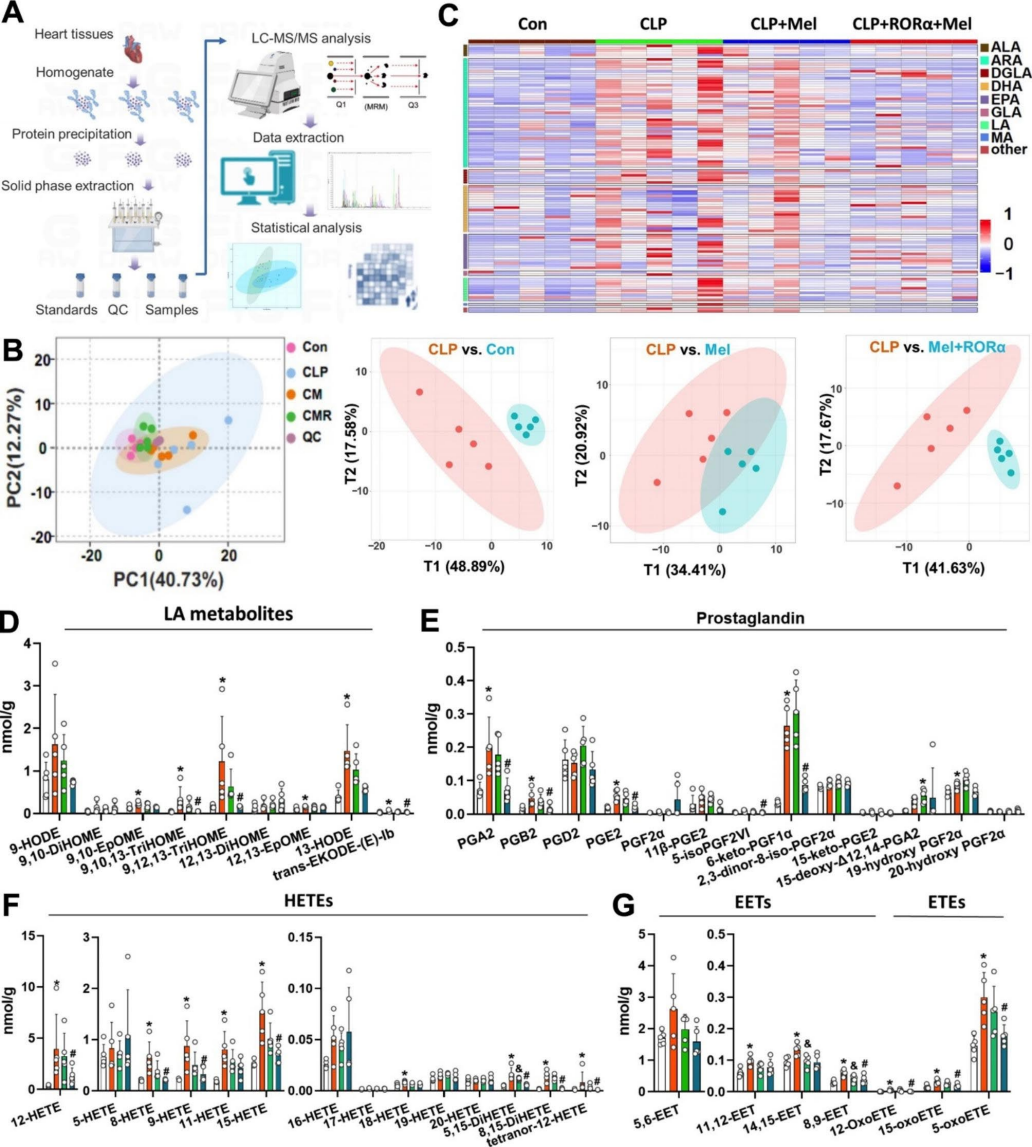
Oxylipin profiles in the sepsis heart were improved by UTMD-mediated RORα overexpression, not by a mild dose of melatonin supplementation alone. (A) Experimental scheme for oxylipin analysis. (B) Principal component analysis (PCA) and partial least squares-discriminant analysis (PLS-DA) showing cardiac oxylipin profiles of sepsis and control group, sepsis and sepsis with melatonin treatment, and sepsis and sepsis with RORα delivery and melatonin treatment. (C) Heat map showing cardiac levels of overall oxylipins across four groups: control, sepsis, sepsis with melatonin treatment, sepsis with RORα delivery, and melatonin treatment. Downregulation and up-regulation are presented in blue and red, respectively. (D) Oxidized products derived from linoleic acid (LA). (E) Oxidized metabolites of arachidonic acid (ARA)

Lipid peroxidation products formed through enzymatic and non-enzymatic reactions were involved in cardiac oxidative injury [31]. We further analyzed 148 polyunsaturated fatty acids (PUFAs) and oxidized products in heart samples (Fig. 7A). A significant difference in oxylipin profiles was noted in sepsis heart after treatment with melatonin and RORα delivery, while the difference was minor when using melatonin monotherapy (Fig. 7B). After excluding metabolites with very low levels due to poor detection accuracy, 108 oxidized products were screened (Fig. 7C). Sepsis moderately decreased PUFAs contents compared to the control (Figure S9), while the lipid peroxidation metabolites in sepsis hearts, especially those derived from arachidonic acid (ARA) and linoleic acid (LA), were substantially counteracted by melatonin and RORα delivery, not melatonin alone (Fig. 7D-E). For example, CLP significantly induced an increase in 8-, 9-, 12-, 15-, Di-hydroxy eicosatetraenoic acids (HETE) and oxo-eicosatetraenoic acids (oxo-ETE), oxidized products of ARA mediated by lipoxygenases, as well as several prostaglandins, while melatonin and RORα delivery significantly counteracted those changes. The antioxidant role of moderate doses of melatonin seemed to depend on the RORα-mediated pathway rather than the direct neutralization.

## Discussion

This study dedicated to deciphering the mechanisms underlying the suboptimal benefits of melatonin in clinical trials confirmed that the nuclear receptor RORα-dependent signal was indispensable for the cardioprotection of a clinically safe dose of melatonin under lethal sepsis. Cardiac RORα delivery via UTMD/CMBs system strikingly optimized the antioxidative effect of melatonin in rats with fatal sepsis beyond a safe dose of melatonin alone. Our findings provide novel insights into the translational dilemma of melatonin in clinic that insufficient receptors may limit melatonin’s effects on myocardial injury.

Numerous experiments have reported promising melatonin therapy in cardiometabolic disease [9, 15, 16, 34–36], while we observed a moderate effect of melatonin treatment on sepsis hearts. Notably, the dose of melatonin (2.5 mg/kg) we used was significantly lower than previous experiments (20–30 mg/kg)[9, 15, 16]. Although high-dose melatonin may be beneficial in neutralizing oxyradical, the potential safety concerns necessitate caution [17]. Raymond and colleagues reported that high-dose intravenous melatonin administration (15 mg/kg) influenced blood cell profiles, and increased plasma creatinine and LDH, although there was no pathological injury in brain, kidney, liver, and other organs [17]. Moreover, recent autopsy analyses found extremely high concentrations of melatonin in blood (ng/ml)[18, 19]. Overall, the effect of melatonin mainly relied on the receptor-dependent pathway [7]. The evidence to support the clinical use of high-dose melatonin was insufficient which may not be feasible in patients. Our study provides translational evidence to optimize the efficacy of a clinically safe dose of melatonin by using UTMD technology.

FDA has approved the use of microbubble technology with C_3_F_8_ as the core gas that supports its clinical safety [27, 37]. Recently, UTMD technology has been used as a therapeutic tool in clinical trials [21, 24]. Compared to traditional microbubbles, CMBs, containing cationic lipids in the membrane have an excellent affinity to anionic DNA and thus promote the delivery efficacy of gene [22]. The liver and spleen have the largest reticuloendothelial system (RES) for phagocytosis of circulating residual CMBs/RORα, which may account for the minor increase of RORα expression in spleen and liver [38]. As observed in our results, the expression of cardiac RORα almost doubled through the target blasting of CMBs. Cardiac gene delivery via UTMD technology has good feasibility and promising clinical translation prospects [22, 23, 39]. Indeed, translational research in primates has shown a great promise of gene delivery by UTMD on the regeneration of islets [40]. Although our previous studies and other demonstrated the favorable effect of a large dose of melatonin on cardiovascular system [9, 10, 15], clinical translation was not satisfactory. Our findings introduced a feasible approach via UTMD-mediated delivery of nuclear receptor to solve the suboptimal effect of clinical dose of melatonin treatment. RORα/CMBs and regular dose of melatonin synergistic therapy effectively alleviated the myocardial oxidative stress and mitochondrial impairment induced by sepsis, and thus significantly improved the heart function and survival status.

Melatonin doses in clinical studies were largely lower than the commonly used in animal studies [13, 41]. The common dose of melatonin in adults was 3–10 mg/day, and the largest dose seemed 60 mg/day for patients with sepsis [42]. This may be one of the key reasons for the inconsistency from basic research to the clinic. As mentioned before [9, 15, 16], the 20–30 mg/kg/day dose of melatonin used in most animal studies was equivalent to a dose of 160–240 mg/day to 60 kg adults [43]. In our study, intravenous melatonin of 5 mg/kg/day to rats was in line with clinical practice (equivalent to a dose of 40–50 mg for 60 kg adults). CMBs had a strong affinity with negatively charged plasmid and UTMD had been applied in clinic. We combined the advantages of both technologies to deliver gene safely and effectively. Our findings confirmed that UTMD-mediated cardiac delivery of melatonin receptors was a promising interdisciplinary strategy to promote the clinical translation of melatonin therapy.

Nuclear receptor RORα is a transcription factor involved in many physiological processes, including metabolism, immunity, and circadian rhythm [6, 44]. Prior studies demonstrated that decreased RORα in ischemic and hypertrophic heart disease might compromise the effect of melatonin [6, 45, 46], while its role in the sepsis heart was unknown. We found that the cardiac expression of RORα, not MT1/2, significantly decreased in rats with lethal sepsis. Our findings provided initial evidence that RORα mediated the cardioprotection of melatonin against fetal sepsis-induced cardiomyopathy, partly by regulating the balance of oxidant and antioxidant signals, including increased Nrf2, SOD1, and MnSOD/SOD2, as well as decreased NOX2/4 and lipid peroxidation. Notably, whether RORs were nuclear melatonin receptor remains controversial due to the lack of strong evidence of direct binding [8]. Our findings emphasized that a clinically safe dose of melatonin alone had limited cardiac benefits under severe sepsis, and the indispensable role of RORα in melatonin therapy should be considered in the clinic.

We observed that sepsis increased circulating inflammation markers, such as TNFα and IL-1β, as well as mitochondrial oxidative stress in heart tissue. Other and our previous studies supported that mitochondrion was the key regulatory center located at the crossroads of pathophysiological processes which seems a promising target to treat cardiopathy [29, 47, 48]. Interestingly, UTMD-mediated cardiac delivery of RORα did not affect inflammation levels but improved mitochondrial dysfunction and oxidative stress. Oxidized lipid products derived from LA and ARA were the most significant metabolites induced by sepsis, which were maximumly improved by ROR delivery and melatonin. Biologically, oxidized products of LA induced mitochondrial dysfunction and the activation of mitochondrial apoptosis signal [49]. Clinical evidence from our previous studies also demonstrated that mitochondria-related metabolite was closely associated with mitochondrial dysfunction [47, 50]. Thus, this study suggested that melatonin activating ROR-dependent pathways improved myocardial redox disorder and oxylipin profiles to alleviate mitochondrial injury assisted by UTMD technology.

The septic cardiomyopathy in this study was based on LPS- and CLP-induced fatal sepsis, with a 48-hour mortality rate of more than 80%. Although gene intervention precedes sepsis modeling in most prior studies [51, 52], the benefits of combining melatonin administration with gene delivery during the clinical course of patients with sepsis require further evaluation. Our study highlights that insufficient expression of RORα may be responsible for the poor efficacy of melatonin at clinical doses. UTMD-mediated gene delivery is a safe and reliable strategy to solve this problem. Second, although we found that the decrease in RORα expression was more pronounced than that of MT1/2 or RORβ/γ in sepsis heart and demonstrated the synergistic action between RORα expression and melatonin administration, the influence of other receptors on melatonin treatment could not be excluded, which requires additional research. Third, we observed that melatonin/RORα signal mediated the recovery of redox balance and improved oxylipin profiles and mitochondrial dysfunction. However, the specific mechanisms in the process need further illustration.

## Conclusion

In summary, our study found that nuclear receptor RORα but not traditional melatonin receptors MT1/2 significantly decreased in sepsis-induced cardiomyopathy. We further designed a UTMD delivery system based on CMBs to assist clinically equivalent dose of melatonin against sepsis-related myocardial injury. UTMD-mediated cardiac delivery of RORα optimized protective effects of melatonin on the septic heart. Insufficient receptors may limit the benefits of melatonin administration and should be taken seriously in the clinic. Our findings may provide distinctive insights and solutions to the suboptimal efficacy of melatonin treatment in clinical trials. Considering the biocompatibility, safety, and efficiency of UTMD technology, the synergistic therapy of melatonin treatment and RORα/CMBs may be a promising therapeutic pattern against sepsis-induced cardiomyopathy.

## Electronic supplementary material

Below is the link to the electronic supplementary material.

Supplementary Material 1

Supplementary Material 2

Supplementary Material 3

Supplementary Material 4

Supplementary Material 5

## Data Availability

The data and materials that support the findings of this study are available from the corresponding author upon reasonable request.

## Abbreviations

4-HNE: 4-hydroxynonenal
ARA: Arachidonic acid
CLP: Cecal ligation and puncture
CMBs: Cationic microbubbles
DC-Chol: 3-[N- (N,N-dimethyl-aminoethane)-carbamoyl
DPPC: 1,2-Dipalmitoyl-sn-glycero-3-phosphocholine
DPPH: 2,2-diphenyl-1-picrylhydrazyl
DSPE-PEG2000: 1,2-distearoyl-sn-glycero-3-phosphoethanolamine-N- [methoxy(polyethylene glycol)-2000]
ECG: Electrocardiograph
H&E: Hematoxylin and eosin
HETE: Hydroxyeicosatetraenoic acid
i.p.: Intraperitoneal
LA: Linoleic acid
LDH: Lactate dehydrogenase
LPS: Lipopolysaccharide
LVEF: Left ventricular ejection fraction
LVFS: Left ventricular fraction shortening
MRM: Multiple reaction monitoring
oxo-ETE: Oxo-eicosatetraenoic acid
PCA: Principal component analysis
PLS-DA: Partial least squares-discriminant analysis
PUFAs: Polyunsaturated fatty acids
RES: Reticuloendothelial system
ROR: Retinoid related orphan nuclear receptor
ROS: Reactive oxygen species
TUNEL: Terminal deoxynucleotidyl transferase-mediated dUTP nick end labeling
UTMD: Ultrasound-targeted microbubble destruction
