# Supplementary material for: Cardiac-targeted delivery of nuclear receptor RORα via ultrasound targeted microbubble destruction optimizes the benefits of regular dose of melatonin on sepsis-induced cardiomyopathy
Source: Biomater Res. 2023 May 5;27:41. doi: 10.1186/s40824-023-00377-8 (PMC10163781; doi:10.1186/s40824-023-00377-8)
Supplement: Supplementary file 5 — Supplementary Material 5 [file 40824_2023_377_MOESM5_ESM.docx]

# Supplementary Figures and Tables

## Figure S1. Serum endotoxin levels increased in rats with CLP surgery

Serum endotoxin was measured 20 hours after CLP surgery using the Limulus test assay. The plasma of the model group was diluted 10-fold and the detection range was 0.1-10 EU/mL. Below 0.1 or above 10 EU/mL were defined as 0.1 and 10 EU/mL, respectively.

Serum endotoxin levels were presented as median (interquartile range). Differences between groups were compared using the rank sum test.

## Figure S2. Isotype control for immunohistochemical staining of cardiac RORα


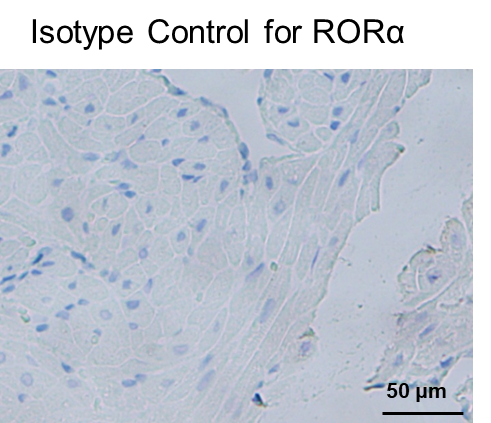


## Figure S3. Melatonin receptors in sepsis-related mRNA microarray or sequencing in GEO database


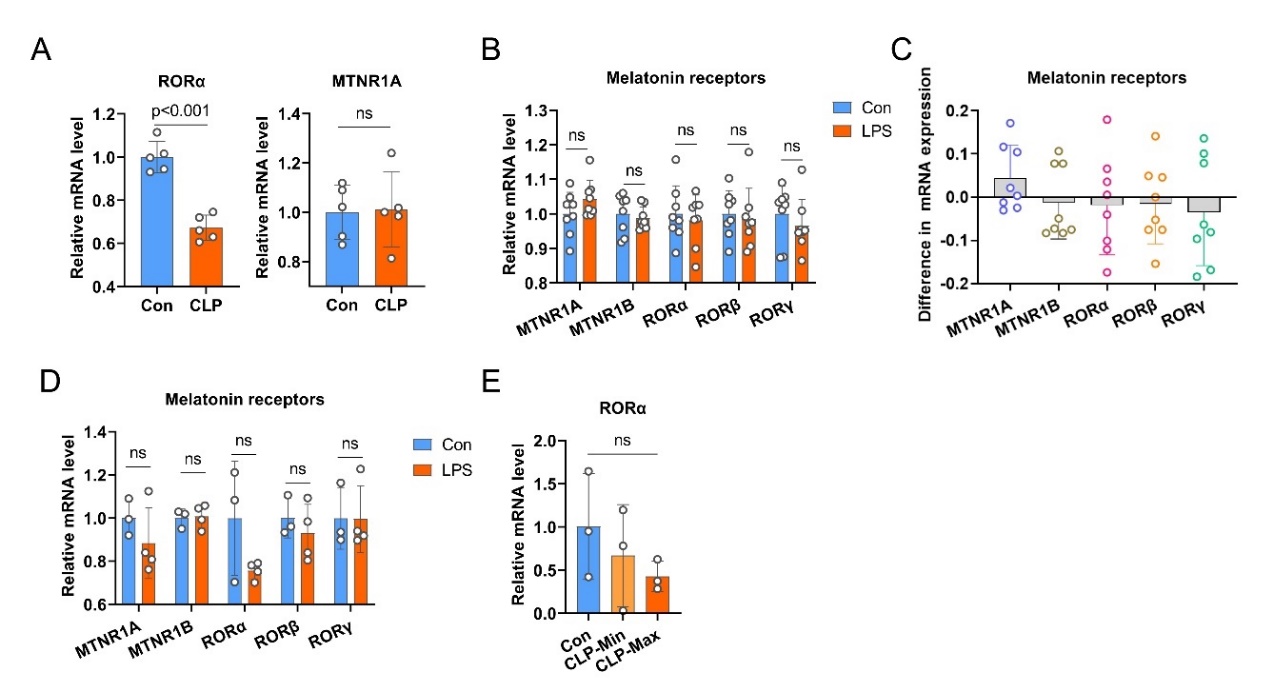


We screened the five melatonin receptors (MTNR1A, MTNR1B, RORα, RORβ, and RORγ) from sepsis-related mRNA microarray or sequencing of GEO database (GEO3037, GEO4479, GSE9667 and GEO56584) under sepsis conditions.

A) mRNA levels of melatonin receptors in lymphocytes from sham or CLP group (n=5). B) mRNA levels of melatonin receptors in peripheral blood neutrophils from septic patients with or without LPS treatment (n=8).

C) difference in the expressions of melatonin receptors in pairs of neutrophils after LPS treatment or not (n=8).

D) mRNA levels of melatonin receptors in neonatal cardiomyocytes with LPS treatment (n=3).

E) Relative mRNA levels of cardiac RORα in sham, and CLP model with mild or severe injury (n=3).

## Figure S4. Characterization of CMBs after binding plasmid


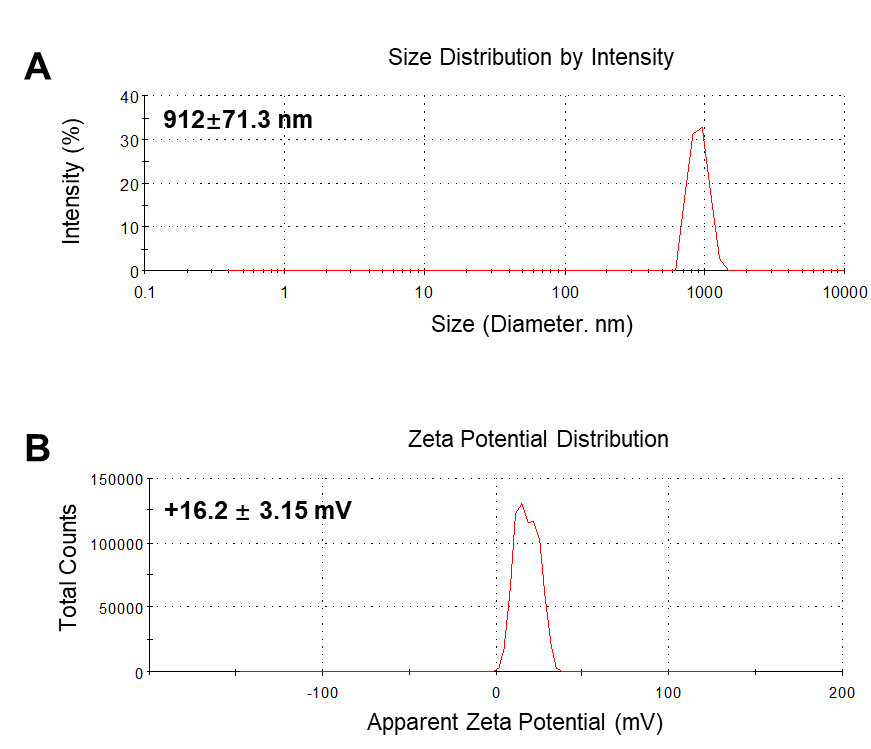


The Size (A) and Zeta potential (B) of the CMBs after binding plasmid.

## Figure S5. Stability of the synthesized CMBs containing plasmid


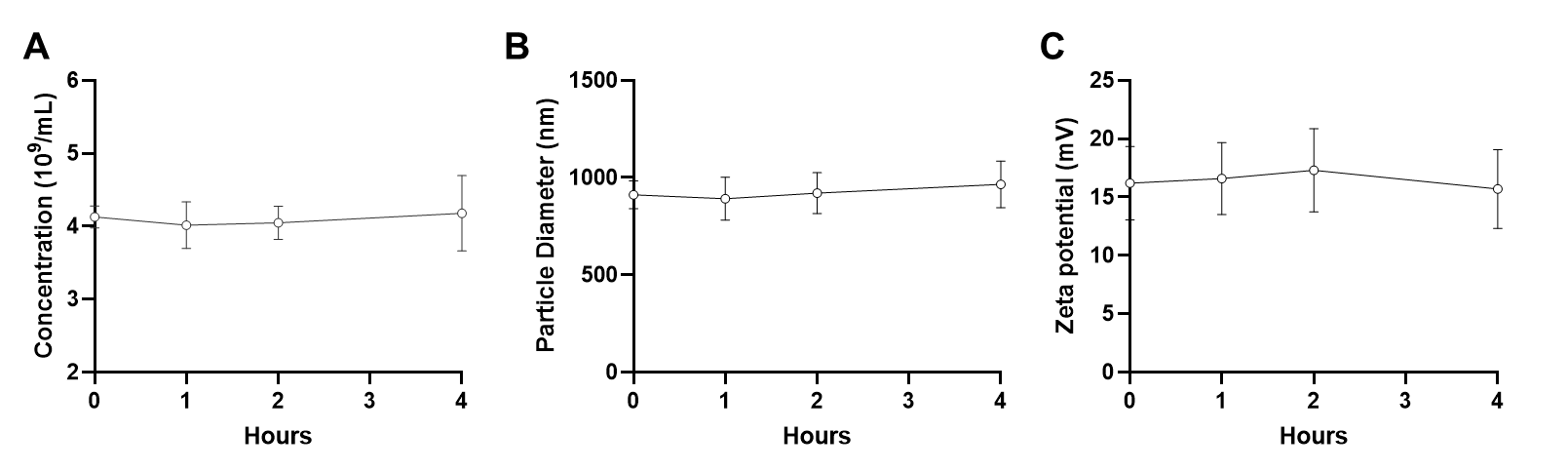


Concentration (A), size (B) and Zeta potential (C) of the CMBs containing plasmid during 4 hours (0/1/2/4) at room temperature (n=4).

## Figure S6. The safety assessment of CMBs injection and UTMD in vivo


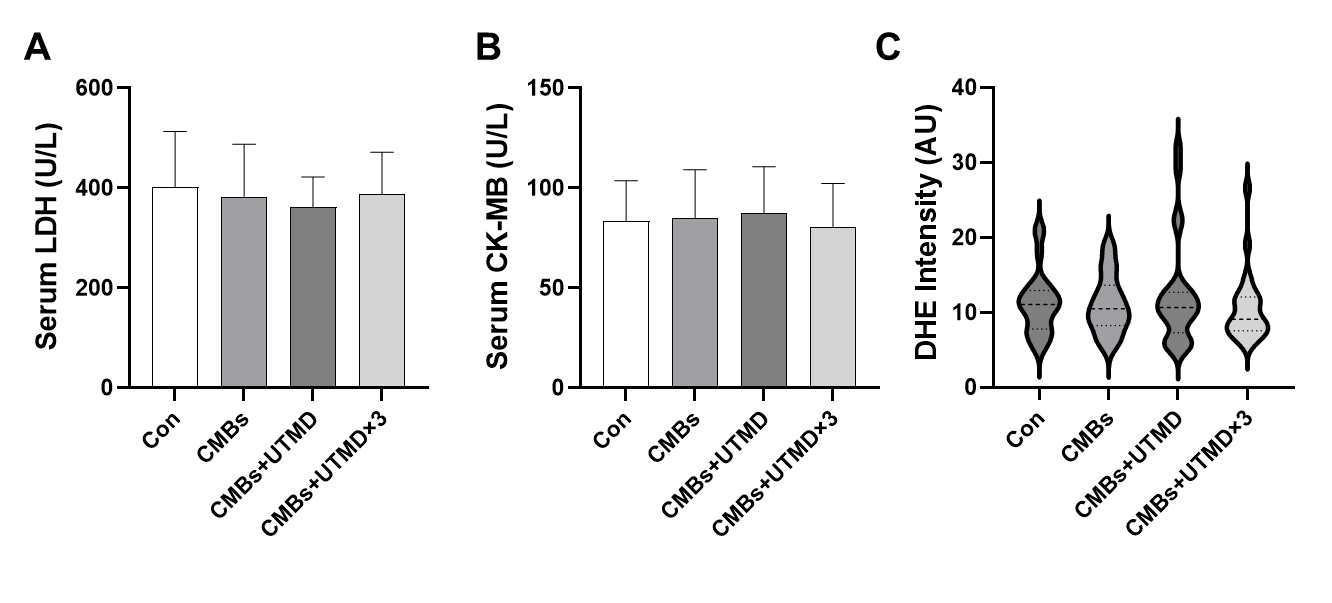


A) Serum level of LDH (a marker of cellular injury) and B) CK-MB (a marker of myocardial injury) were evaluated using commercial kits before and after CMBs injection, UTMD treatment (n = 5). C) DHE intensity was quantified to reflect myocardial ROS levels (n = 5, ~25 views per group). CMBs+UTMD×3 group was given 3 repeated UTMD treatments at one-day intervals 1 day, 3 days, and 5 days. Blood and heart tissues were collected 6 hours after operation according to the characteristics of biomarker leak after myocardial injury.

## Figure S7. UTMD and CMBs did not affect the antioxidant activity of the melatonin in vitro

DPPH assay was used to measure the antioxidant activity in vitro (n=5). Sample without DPPH were set as the blank control. The absorbance was measured at 515 nm using Spectrophotometer. Antioxidant activity was calculated according to the manufacturers' instructions and expressed in percentage.

## Figure S8. Isotype control for immunohistochemical staining of cardiac 4-HNE


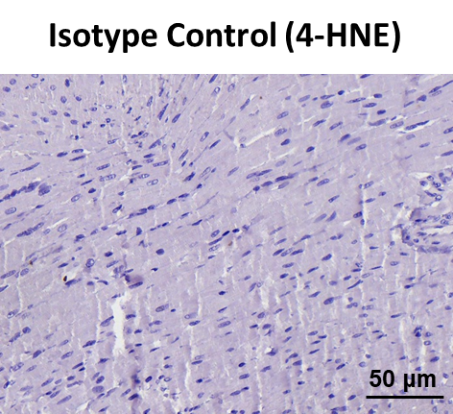


## Figure S9. Cardiac contents of polyunsaturated fatty acids and oxidative products of α-Linolenic acid


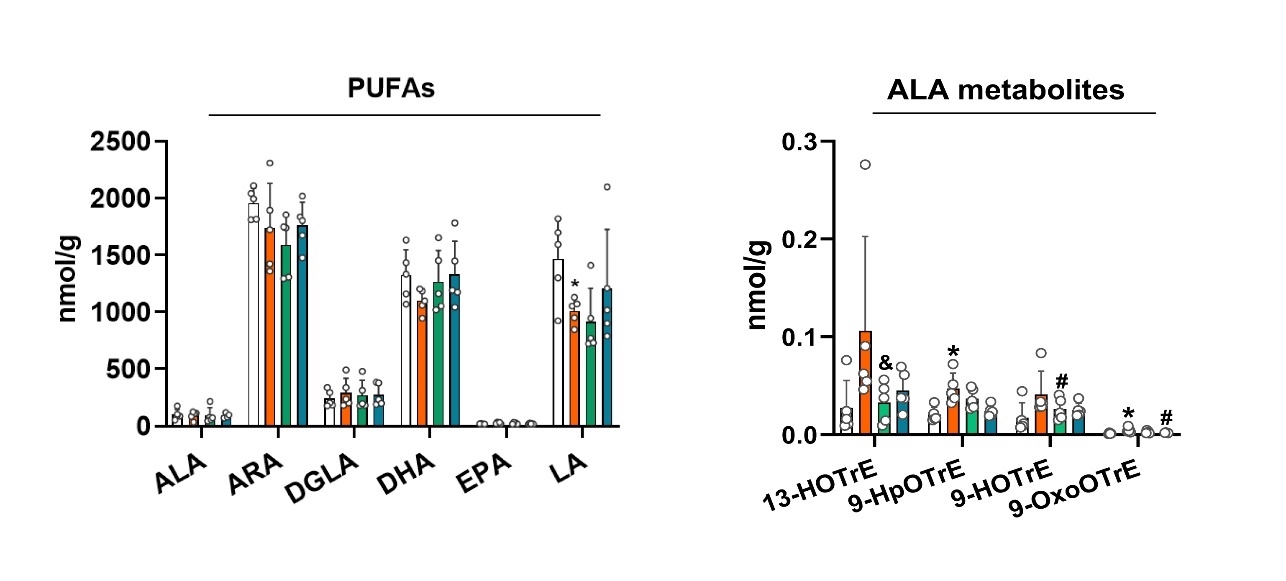


Several PUFAs, the sources of oxidized lipid metabolites in heart tissues, and α-linolenic acid (ALA) were detected in septic heart after 20 hours CLP surgery.

## Table S1. List of RT-qPCR primers

| **Gene name** | **Sequence (5'to3')** | |
| --- | --- | --- |
| MTNR1A | Forward | CATAGCCATCATGCCCAACC |
|  | Reverse | TCCGTCTGACCTGAAGAACC |
| MTNR1B | Forward | AACTTCCGCAGGGAGTACAA |
|  | Reverse | TCCTCAGTCAGGTGGCATTT |
| RORα | Forward | AATGCCACCTACTCCTGTCC |
|  | Reverse | GCGTACAGGCTGTCTCTTTG |
| RORβ | Forward | GAGGAGCCAGCAGAACAATG |
|  | Reverse | GCTTCTGCACCTCAGCATAC |
| RORγ | Forward | CAGTGCAATGTGGCCTACTC |
|  | Reverse | CTGTCCCTCTGCTTCTTGGA |

## Table S2. List of primary antibodies

| **Antibodies** | **Source** | **Identifier** | **Dilution** |
| --- | --- | --- | --- |
| 4-HNE | R&D | Cat#:MAB3249 | IHC 0.1ug/mL |
| GAPDH | ZSGB-BIO | Cat#:ta-08 | WB 1:5000 |
| HO-1 | Abcam | Cat#: ab83214 | WB 1:1000 |
| KEAP1 | Proteintech | Cat#: 10503-2-AP | WB 1:2000 |
| MTNR1A | Bioss antibody | Cat#: bs-0027R | WB 1:1000 |
| MTNR1B | Bioss antibody | Cat#: bs-0963R | WB 1:1000 |
| NOX2 | Proteintech | Cat#: 19013-1-AP | WB 1:2000 |
| NOX4 | Bioss antibody | Cat#:bs-1091R | WB 1:1000 |
| NRF2 | Proteintech | Cat#: 16396-1-AP | WB 1:1000 |
| RORα | Abcam | Cat#: ab70061 | WB 1:2000；  IHC 1:200 |
| SOD2 | Proteintech | Cat#: 24127-1-AP | WB 1:10000 |

WB, western blotting. IHC, immunohistochemistry.

## Table S3. Basic information of oxylipin profiles

| **Index** | **Compound name** | **Class** | **RT** | **LLOQ** | **ULOQ** |
| --- | --- | --- | --- | --- | --- |
| mws-oxlipid-A1 | 14,15-EET[(±)14,15-epoxy-5Z,8Z,11Z-eicosatrienoic acid] | ARA | 3.91 | 0.2 | 400 |
| mws-oxlipid-A2 | 11,12-EET[(±)11,12-epoxy-5Z,8Z,14Z-eicosatrienoic acid] | ARA | 4.11 | 0.2 | 400 |
| mws-oxlipid-A3 | 8,9-EET[(±)8,9-epoxy-5Z,11Z,14Z-eicosatrienoic acid] | ARA | 4.18 | 0.2 | 400 |
| mws-oxlipid-A4 | 5,6-EET[(±)5,6-epoxy-8Z,11Z,14Z-eicosatrienoic acid] | ARA | 4.25 | 1 | 400 |
| mws-oxlipid-B1 | 9-oxoODE[9-oxo-10E,12Z-octadecadienoic acid] | LA | 3.17 | 0.2 | 400 |
| mws-oxlipid-B2 | 13-oxoODE[13-oxo-9Z,11E-octadecadienoic acid] | LA | 3.16 | 0.2 | 400 |
| mws-oxlipid-B3 | trans-EKODE-(E)-Ib[9-oxo-11-(3-pentyl-2-oxiranyl)-10E-undecenoic acid] | LA | 2.52 | 0.2 | 400 |
| mws-oxlipid-B4 | 13(S)-HODE[13S-hydroxy-9Z,11E-octadecadienoic acid] | LA | 3.03 | 0.2 | 400 |
| mws-oxlipid-B5 | (±)9-HODE[(±)-9-hydroxy-10E,12Z-octadecadienoic acid] | LA | 3.05 | 0.2 | 400 |
| mws-oxlipid-B6 | 13(S)-HpODE[13S-hydroperoxy-9Z,11E-octadecadienoic acid] | LA | 3.18 | 4 | 400 |
| mws-oxlipid-C1 | (±)18-HEPE[(±)-18-hydroxy-5Z,8Z,11Z,14Z,16E-eicosapentaenoic acid] | EPA | 2.51 | 0.4 | 400 |
| mws-oxlipid-C2 | (±)15-HEPE[(±)-15-hydroxy-5Z,8Z,11Z,13E,17Z-eicosapentaenoic acid] | EPA | 2.68 | 0.2 | 400 |
| mws-oxlipid-C3 | (±)12-HEPE[(±)-12-hydroxy-5Z,8Z,10E,14Z,17Z-eicosapentaenoic acid] | EPA | 2.81 | 0.2 | 400 |
| mws-oxlipid-C4 | (±)5-HEPE[(±)-5-hydroxy-6E,8Z,11Z,14Z,17Z-eicosapentaenoic acid] | EPA | 2.92 | 0.2 | 400 |
| mws-oxlipid-C5 | (±)11-HEPE[(±)-11-hydroxy-5Z,8Z,12E,14Z,17Z-eicosapentaenoic acid] | EPA | 2.68 | 1 | 400 |
| mws-oxlipid-C6 | (±)8-HEPE[(±)-8-hydroxy-5Z,9E,11Z,14Z,17Z-eicosapentaenoic acid] | EPA | 2.75 | 0.2 | 400 |
| mws-oxlipid-C7 | (±)9-HEPE[(±)-9-hydroxy-5Z,7E,11Z,14Z,17Z-eicosapentaenoic acid] | EPA | 2.83 | 0.2 | 400 |
| mws--oxlipid-E1 | 20-HETE[20-hydroxy-5Z,8Z,11Z,14Z-eicosatetraenoic acid] | ARA | 2.66 | 0.2 | 400 |
| mws--oxlipid-E10 | (±)5-HETE[(±)5-hydroxy-6E,8Z,11Z,14Z-eicosatetraenoic acid] | ARA | 3.62 | 4 | 4000 |
| mws--oxlipid-E11 | 8-HETE[8-hydroxy-5Z,9E,11Z,14Z-eicosatetraenoic acid] | ARA | 3.42 | 2 | 4000 |
| mws--oxlipid-E12 | tetranor-12(S)-HETE[8S-hydroxy-4Z,6E,10Z-hexadecatrienoic acid] | ARA | 2.06 | 0.2 | 400 |
| mws--oxlipid-E2 | 19(S)-HETE[19S-hydroxy-5Z,8Z,11Z,14Z-eicosatetraenoic acid] | ARA | 2.62 | 0.4 | 400 |
| mws--oxlipid-E3 | (±)18-HETE[(±)18-hydroxy-5Z,8Z,11Z,14Z-eicosatetraenoic acid] | ARA | 2.83 | 0.2 | 400 |
| mws--oxlipid-E4 | (±)17-HETE[(±)17-hydroxy-5Z,8Z,11Z,14Z-eicosatetraenoic acid] | ARA | 2.87 | 0.2 | 400 |
| mws--oxlipid-E5 | (±)16-HETE[(±)16-hydroxy-5Z,8Z,11Z,14Z-eicosatetraenoic acid] | ARA | 2.90 | 0.2 | 400 |
| mws--oxlipid-E6 | (±)15-HETE[(±)15-hydroxy-5Z,8Z,11Z,13E-eicosatetraenoic acid] | ARA | 3.18 | 2 | 4000 |
| mws--oxlipid-E7 | (±)12-HETE[(±)12-hydroxy-5Z,8Z,10E,14Z-eicosatetraenoic acid] | ARA | 3.44 | 2 | 4000 |
| mws--oxlipid-E8 | 11(S)-HETE[11S-hydroxy-5Z,8Z,12E,14Z-eicosatetraenoic acid] | ARA | 3.31 | 2 | 4000 |
| mws--oxlipid-E9 | (±)9-HETE[(±)-9-hydroxy-5Z,7E,11Z,14Z-eicosatetraenoic acid] | ARA | 3.52 | 2 | 4000 |
| mws-oxlipid-F1 | EPA[Eicosapentaenoic Acid] | EPA | 4.89 | 40 | 40000 |
| mws-oxlipid-F2 | ARA[Arachidonic Acid] | ARA | 5.41 | 40 | 40000 |
| mws-oxlipid-F3 | DHA[Docosahexaenoic Acid] | DHA | 5.27 | 40 | 40000 |
| mws-oxlipid-F4 | DGLA[Dihomo-γ-Linolenic Acid] | DGLA | 5.63 | 40 | 40000 |
| mws-oxlipid-F5 | LA[Linoleic Acid] | LA | 5.49 | 40 | 40000 |
| mws-oxlipid-F6 | ALA[α-Linolenic Acid] | ALA | 5.00 | 40 | 40000 |
| mws-oxlipid-F7 | GLA[γ-Linolenic Acid] | GLA | 5.08 | 40 | 40000 |
| mws-oxlipid-F8 | 20-COOH-AA[20-Carboxyarachidonic Acid] | ARA | 2.31 | 0.4 | 400 |
| mws-oxlipid-G1 | (±)4-HDHA/HDoHE[(±)4-hydroxy-5E,7Z,10Z,13Z,16Z,19Z-docosahexaenoic acid] | DHA | 3.75 | 4 | 4000 |
| mws-oxlipid-G10 | (±)20-HDHA/HDoHE[(+/-)20-Hydroxy-4Z,7Z,10Z,13Z,16Z,18E-Docosahexaenoic Acid] | DHA | 3.05 | 2 | 4000 |
| mws-oxlipid-G2 | (±)7-HDHA/HDoHE[(±)7-hydroxy-4Z,8E,10Z,13Z,16Z,19Z-docosahexaenoic acid] | DHA | 3.45 | 4 | 4000 |
| mws-oxlipid-G3 | (±)14-HDHA/HDoHE[14S-hydroxy-4Z,7Z,10Z,12E,16Z,19Z-docosahexaenoic acid] | DHA | 3.31 | 4 | 4000 |
| mws-oxlipid-G4 | (±)17-HDHA/HDoHE[(±)17-hydroxy-4Z,7Z,10Z,13Z,15E,19Z-docosahexaenoic acid] | DHA | 3.20 | 4 | 4000 |
| mws-oxlipid-G5 | (±)13-HDHA/HDoHE[(+/-)-13-hydroxy-4Z,7Z,10Z,14E,16Z,19Z-docosahexaenoic acid] | DHA | 3.23 | 1 | 4000 |
| mws-oxlipid-G6 | (±)10-HDHA/HDoHE[ (+/-)-10-Hydroxy-4Z,7Z,11E,13Z,16Z,19Z-Docosahexaenoic Acid] | DHA | 3.31 | 1 | 4000 |
| mws-oxlipid-G7 | (±)8-HDHA/HDoHE[(+/-)-8-Hydroxy-4Z,6E,10Z,13Z,16Z,19Z-Docosahexaenoic Acid] | DHA | 3.50 | 1 | 4000 |
| mws-oxlipid-G8 | (±)11-HDHA/HDoHE[(+/-)-11-Hydroxy-4Z,7Z,9E,13Z,16Z,19Z-Docosahexaenoic Acid] | DHA | 3.39 | 1 | 4000 |
| mws-oxlipid-G9 | (±)16-HDHA/HDoHE[(+/-)-16-Hydroxy-4Z,7Z,10Z,13Z,17E,19Z-Docosahexaenoic Acid] | DHA | 3.17 | 1 | 4000 |
| mws-oxlipid-H1 | RvD3[4S,11R,17S-trihydroxy-5Z,7E,9E,13Z,15E,19Z-docosahexaenoic acid] | DHA | 0.96 | 0.2 | 400 |
| mws-oxlipid-H2 | RvD2[7S,16R,17S-trihydroxy-4Z,8E,10Z,12E,14E,19Z-docosahexaenoic acid] | DHA | 0.98 | 0.2 | 400 |
| mws-oxlipid-H3 | RvD1[7S,8R,17S-trihydroxy-4Z,9E,11E,13Z,15E,19Z-docosahexaenoic acid] | DHA | 1.08 | 0.2 | 400 |
| mws-oxlipid-H4 | RvD5[7S,17S-dihydroxy-4Z,8E,10Z,13Z,15E,19Z-docosahexaenoic acid] | DHA | 1.63 | 0.2 | 400 |
| mws-oxlipid-J1 | PGE1[9-oxo-11α,15S-dihydroxy-prost-13E-en-1-oic acid] | DGLA | 0.96 | 0.2 | 400 |
| mws-oxlipid-J2 | PGD1[9α,15S-dihydroxy-11-oxo-prost-13E-en-1-oic acid] | DGLA | 1.01 | 0.2 | 400 |
| mws-oxlipid-JJ1 | PGE2[9-oxo-11α,15S-dihydroxy-prosta-5Z,13E-dien-1-oic acid] | ARA | 0.98 | 0.2 | 400 |
| mws-oxlipid-JJ2 | PGD2[9α,15S-dihydroxy-11-oxo-prosta-5Z,13E-dien-1-oic acid] | ARA | 1.00 | 0.2 | 400 |
| mws-oxlipid-K1 | PGF3α[9α,11α,15S-trihydroxy-prosta-5Z,13E,17Z-trien-1-oic acid] | EPA | 0.82 | 10 | 400 |
| mws-oxlipid-K10 | PGA2[9-oxo-15S-hydroxy-5Z,10Z,13E-prostatrienoic acid] | ARA | 1.44 | 0.4 | 400 |
| mws-oxlipid-K11 | PGB2[15S-hydroxy-9-oxo-5Z,8(12),13E-prostatrienoic acid] | ARA | 1.37 | 0.4 | 400 |
| mws-oxlipid-K12 | PGD3[9S,15S-dihydroxy-11-oxo-5Z,13E,17Z-prostatrienoic acid] | EPA | 0.93 | 0.4 | 400 |
| mws-oxlipid-K13 | 15-keto-PGF1α[9S,11R-dihydroxy-15-oxo-13E-prostaenoic acid] | DGLA | 1.01 | 4 | 400 |
| mws-oxlipid-K14 | 15-keto-PGF2α[9S,11R-dihydroxy-15-oxo-5Z,13E-prostadienoic acid] | ARA | 1.00 | 4 | 400 |
| mws-oxlipid-K15 | 15-keto-PGE2[9,15-dioxo-11R-hydroxy-5Z,13E-prostadienoic acid] | ARA | 1.06 | 0.4 | 400 |
| mws-oxlipid-K16 | 13,14-dihydro-15-keto PGD2[11,15-dioxo-9S-hydroxy-5Z-prostenoic acid] | ARA | 1.30 | 0.4 | 400 |
| mws-oxlipid-K17 | 13,14-dihydro-15-keto PGE2[9,15-dioxo-11R-hydroxy-5Z-prostenoic acid] | ARA | 1.30 | 4 | 400 |
| mws-oxlipid-K18 | 8-iso-PGF2α[8-iso 9α,11α,15S-trihydroxy-prosta-5Z,13E-dien-1-oic acid] | ARA | 0.83 | 10 | 400 |
| mws-oxlipid-K19 | 2,3-dinor-8-iso-PGF2α[2,3-Dinor-8-Epi-Prostaglandin F2alpha] | ARA | 0.74 | 2 | 400 |
| mws-oxlipid-K2 | 6-keto-PGF1α[6 keto-PGF1α[6-oxo-9α,11α,15S-trihydroxy-prost-13E-en-1-oic acid] | ARA | 0.78 | 0.2 | 400 |
| mws-oxlipid-K20 | 12-HHT[12-Hydroxy-5,8,10-heptadecatrienoic acid] | ARA | 2.12 | 0.4 | 400 |
| mws-oxlipid-K21 | tetranor-PGFM[tetranor-PGFM] | ARA | 0.91 | 0.4 | 400 |
| mws-oxlipid-K22 | 15-deoxy-Δ12,14-PGA2[15-deoxy-Δ12,14-PGA2[9-oxo-prosta-5Z,10,12Z,14E-tetraen-1-oic acid] | ARA | 2.65 | 0.2 | 400 |
| mws-oxlipid-K23 | 13,14-dihydro-15-keto PGF2α[9α,11α-dihydroxy-15-oxo-prost-5Z-en-1-oic acid] | ARA | 1.13 | 4 | 400 |
| mws-oxlipid-K24 | 13,14-dihydro PGF2α[9α,11α,15S-trihydroxy-prost-5Z-en-1-oic acid] | ARA | 1.01 | 4 | 400 |
| mws-oxlipid-K25 | 19(R)-hydroxy PGF2α[9α,11α,15S,19R-tetrahydroxy-prosta-5Z,13E-dien-1-oic acid] | ARA | 0.76 | 1 | 400 |
| mws-oxlipid-K26 | 20-hydroxy PGF2α[9α,11α,15S,20-tetrahydroxy-prosta-5Z,13E-dien-1-oic acid] | ARA | 0.72 | 1 | 400 |
| mws-oxlipid-K3 | PGF2α[9α,11α,15S-trihydroxy-prosta-5Z,13E-dien-1-oic acid] | ARA | 1.00 | 0.2 | 400 |
| mws-oxlipid-K4 | PGF1α[9α,11α,15S-trihydroxy-prost-13E-en-1-oic acid] | DGLA | 0.88 | 0.2 | 400 |
| mws-oxlipid-K5 | PGK1[9,11-dioxo-15S-hydroxy-prost-13E-en-1-oic acid] | DGLA | 0.92 | 0.2 | 400 |
| mws-oxlipid-K6 | PGJ2[11-oxo-15S-hydroxy-prosta-5Z,9,13E-trien-1-oic acid] | ARA | 1.37 | 0.4 | 400 |
| mws-oxlipid-K7 | 11-deoxy PGE1[9-oxo-15S-hydroxy-prost-13E-en-1-oic acid] | DGLA | 1.62 | 0.2 | 400 |
| mws-oxlipid-K8 | 11β-PGE2[9-oxo-11S,15S-dihydroxy-5Z,13E-prostadienoic acid] | ARA | 0.97 | 0.4 | 400 |
| mws-oxlipid-K9 | 11β-PGF2α[9S,11S,15S-trihydroxy-5Z,13E-prostadienoic acid] | ARA | 0.83 | 1 | 400 |
| mws-oxlipid-L1 | RvE1[5S,12R,18R-trihydroxy-6Z,8E,10E,14Z,16E-eicosapentaenoic acid] | EPA | 0.77 | 0.2 | 400 |
| mws-oxlipid-L10 | LTE4[5S-hydroxy-6R-(S-cysteinyl)-7E,9E,11Z,14Z-eicosatetraenoic acid] | ARA | 1.09 | 1 | 400 |
| mws-oxlipid-L11 | LTB4[5S,12R-dihydroxy-6Z,8E,10E,14Z-eicosatetraenoic acid] | ARA | 1.67 | 0.2 | 400 |
| mws-oxlipid-L12 | 11-keto-TXB2[9S,15S-Dihydroxy-11-Oxo-Thromboxa-5Z,13E-Dien-1-Oic Acid] | ARA | 0.97 | 0.2 | 400 |
| mws-oxlipid-L13 | TXB1[9S,11,15S-trihydroxy-thrombox-13E-enoic acid] | DGLA | 0.80 | 0.2 | 400 |
| mws-oxlipid-L14 | 20-OH-LTB4[5S,12R,20-trihydroxy-6Z,8E,10E,14Z-eicosatetraenoic acid] | ARA | 0.77 | 0.2 | 400 |
| mws-oxlipid-L15 | 20-COOH-LTB4[5S,12R-dihydroxy-6Z,8E,10E,14Z-eicosatetraene-1,20-dioic acid] | ARA | 0.76 | 10 | 400 |
| mws-oxlipid-L16 | 6-trans-LTB4[5S,12R-dihydroxy-6E,8E,10E,14Z-eicosatetraenoic acid] | ARA | 1.61 | 0.1 | 400 |
| mws-oxlipid-L17 | 11-trans-LTE4[5S-hydroxy-6R-(S-cysteinyl)-7E,9E,11E14Z-eicosatetraenoic acid] | ARA | 1.09 | 0.2 | 400 |
| mws-oxlipid-L18 | 14,15-LTE4[ 15S-hydroxy,14R-(S-cysteinyl)-5Z,8Z,10E,12E-eicosatetraenoic acid] | ARA | 1.03 | 4 | 400 |
| mws-oxlipid-L2 | TXB2[9α,11,15S-trihydroxythromba-5Z,13E-dien-1-oic acid] | ARA | 0.84 | 1 | 400 |
| mws-oxlipid-L20 | 12-epi LTB4[5S,12S-dihydroxy-6Z,8E,10E,14Z-eicosatetraenoic acid] | ARA | 1.67 | 1 | 400 |
| mws-oxlipid-L21 | 6-trans-12-epi?LTB4[5S,12S-dihydroxy-6E,8E,10E,14Z-eicosatetraenoic acid] | ARA | 1.61 | 1 | 400 |
| mws-oxlipid-L3 | TxB3[9α,11,15S-trihydroxythromba-5Z,13E,17Z-trien-1-oic acid] | EPA | 0.89 | 1 | 400 |
| mws-oxlipid-L4 | LTD4[5S-hydroxy-6R-(S-cysteinylglycinyl)-7E,9E,11Z,14Z-eicosatetraenoic acid] | ARA | 0.83 | 0.4 | 400 |
| mws-oxlipid-L5 | LXA5[5S,6R,15S-trihydroxy-7E,9E,11Z,13E,17Z-eicosapentaenoic acid] | EPA | 0.93 | 0.2 | 400 |
| mws-oxlipid-L6 | LXA4[5S,6R,15S-trihydroxy-7E,9E,11Z,13E-eicosatetraenoic acid] | ARA | 1.06 | 0.2 | 400 |
| mws-oxlipid-L7 | LXB4[5S,14R,15S-trihydroxy-6E,8Z,10E,12E-eicosatetraenoic acid] | ARA | 0.95 | 0.4 | 400 |
| mws-oxlipid-L8 | 1-Mar[7R,14S-dihydroxy-4Z,8E,10E,12Z,16Z,19Z-docosahexaenoic acid] | DHA | 1.60 | 0.2 | 400 |
| mws-oxlipid-L9 | PDX[10(S),17(S)-dihydroxy-4Z,7Z,11E,13Z,15E,19Z-docosahexaenoic acid] | DHA | 1.60 | 0.2 | 400 |
| mws-oxlipid-N1 | 9,10-DiHOME[(±)9,10-dihydroxy-12Z-octadecenoic acid] | LA | 1.94 | 0.2 | 400 |
| mws-oxlipid-N10 | 17(18)-EpETE[(±)17,18-epoxy-5Z,8Z,11Z,14Z-eicosatetraenoic acid] | EPA | 3.24 | 0.2 | 400 |
| mws-oxlipid-N11 | 16(17)-EpDPE[(±)16,17-epoxy-4Z,7Z,10Z,13Z,19Z-docosapentaenoic acid] | DHA | 3.94 | 0.2 | 400 |
| mws-oxlipid-N12 | 5-HETrE[5S-hydroxy-6E,8Z,11Z-eicosatrienoic acid] | MA | 4.40 | 0.2 | 400 |
| mws-oxlipid-N13 | (±)5,6-DIHETE[(±)5,6-dihydroxy-8Z,11Z,14Z,17Z-eicosatetraenoic acid] | EPA | 2.02 | 1 | 400 |
| mws-oxlipid-N14 | 5(S),15(S)-DiHETE[5S,15S-dihydroxy-6E,8Z,10Z,13E-eicosatetraenoic acid] | ARA | 1.60 | 0.2 | 400 |
| mws-oxlipid-N15 | (±)8(9)-DiHETE[(+/-)-8,9-dihydroxy-5Z,11Z,14Z,17Z-eicosatetraenoic acid] | EPA | 1.87 | 0.2 | 400 |
| mws-oxlipid-N16 | (±)11(12)-DiHETE[(+/-)-11,12-dihydroxy-5Z,8Z,14Z,17Z-eicosatetraenoic acid] | EPA | 1.80 | 0.2 | 400 |
| mws-oxlipid-N17 | (±)14(15)-DiHETE[(+/-)-14,15-dihydroxy-5Z,8Z,11Z,17Z-eicosatetraenoic acid] | EPA | 1.77 | 0.2 | 400 |
| mws-oxlipid-N18 | (±)17(18)-DiHETE[(+/-)-17,18-dihydroxy-5Z,8Z,11Z,14Z-eicosatetraenoic acid] | EPA | 1.65 | 0.2 | 400 |
| mws-oxlipid-N19 | (±)14(15)-DiHET[(+/-)14,15-dihydroxy-5Z,8Z,11Z-eicosatrienoic acid] | ARA | 2.09 | 0.2 | 400 |
| mws-oxlipid-N2 | 5-oxoETE[5-oxo-6E,8Z,11Z,14Z-eicosatetraenoic acid] | ARA | 4.01 | 0.2 | 400 |
| mws-oxlipid-N20 | 8(S)-HETrE[8S-hydroxy-9E,11Z,14Z-eicosatrienoic acid] | DGLA | 3.67 | 0.2 | 400 |
| mws-oxlipid-N21 | 15(S)-HETrE[15S-hydroxy-8Z,11Z,13E-eicosatrienoic acid] | DGLA | 3.55 | 0.2 | 400 |
| mws-oxlipid-N22 | (±)19(20)-EpDPE(A)[(+/-)-19(20)-Epoxy-4Z,7Z,10Z,13Z,16Z-Docosapentaenoic Acid] | DHA | 3.75 | 0.2 | 400 |
| mws-oxlipid-N23 | (±)7(8)-DiHDPE(A)[(+/-)-7(8)-dihydroxy-4Z,10Z,13Z,16Z,19Z-Docosapentaenoic Acid] | DHA | 2.56 | 0.2 | 400 |
| mws-oxlipid-N24 | (±)13(14)-DiHDPE(A)[(+/-)-13(14)-dihydroxy-4Z,7Z,10Z,16Z,19Z-Docosapentaenoic Acid] | DHA | 2.26 | 0.2 | 400 |
| mws-oxlipid-N25 | (±)19(20)-DiHDPE(A)[(+/-)-19(20)-dihydroxy-4Z,7Z,10Z,13Z,16Z-Docosapentaenoic Acid] | DHA | 2.05 | 4 | 400 |
| mws-oxlipid-N26 | (±)8(9)-DiHET[8,9-dihydroxy-5Z,11Z,14Z-eicosatrienoic acid] | ARA | 2.43 | 0.2 | 400 |
| mws-oxlipid-N27 | (±)11(12)-DiHET[11,12-dihydroxy-5Z,8Z,14Z-eicosatrienoic acid] | ARA | 2.28 | 0.2 | 400 |
| mws-oxlipid-N28 | 9(S),10(S),13(S)-TriHOME[9（S）,10(S),13(S)-Trihydroxy-11-Octadecenoic Acid] | LA | 0.90 | 0.2 | 400 |
| mws-oxlipid-N29 | 9(S),12(S),13(S)-TriHOME[9S,12S,13S-Trihydroxy-10E-Octadecenoic Acid] | LA | 0.91 | 0.4 | 400 |
| mws-oxlipid-N3 | 15-oxoETE[15-oxo-5Z,8Z,11Z,13E-eicosatetraenoic acid] | ARA | 3.37 | 0.2 | 400 |
| mws-oxlipid-N30 | (±)14(15)-EpETE[(+/-)-14(15)-Epoxy-5Z,8Z,11Z,17Z-Eicosatetraenoic Acid] | EPA | 3.40 | 0.2 | 400 |
| mws-oxlipid-N31 | 11-HEDE[(+/-)11-Hydroxy-12E,14Z-Eicosadienoic Acid] | - | 4.08 | 0.2 | 400 |
| mws-oxlipid-N32 | 15-HEDE[(+/-)-15-Hydroxy-11Z,13E-Eicosadienoic Acid] | - | 4.10 | 0.2 | 400 |
| mws-oxlipid-N33 | 9-OxoOTrE[9-oxo-10E,12Z,15Z-octadecatrienoic acid] | ALA | 2.79 | 0.2 | 400 |
| mws-oxlipid-N34 | 13(S)-HOTrE(γ)[13S-hydroxy-6Z,9Z,11E-octadecatrienoic acid] | GLA | 2.60 | 0.2 | 400 |
| mws-oxlipid-N35 | 9(S)-HpOTrE[9S-hydroperoxy-10E,12Z,15Z-octadecatrienoic acid] | ALA | 2.51 | 0.2 | 400 |
| mws-oxlipid-N36 | (±)12(13)-DiHOME[12,13-dihydroxy-9Z-octadecenoic acid] | LA | 1.84 | 0.2 | 400 |
| mws-oxlipid-N37 | 12-OxoETE[12-oxo-5Z,8Z,10E,14Z-eicosatetraenoic acid] | ARA | 3.45 | 4 | 400 |
| mws-oxlipid-N38 | 5(S),12(S)-DiHETE[5S,12S-dihydroxy-6E,8Z,10E,14Z-eicosatetraenoic acid] | ARA | 1.87 | 1 | 400 |
| mws-oxlipid-N39 | 8(S),15(S)-DiHETE[8S,15S-dihydroxy-5Z,9E,11Z,13E-eicosatetraenoic acid] | ARA | 1.53 | 0.2 | 400 |
| mws-oxlipid-N4 | 5,6-DiHETrE[(±)5,6-dihydroxy-8Z,11Z,14Z-eicosatrienoic acid] | ARA | 2.64 | 0.2 | 400 |
| mws-oxlipid-N40 | (±)7,8-EpDPE[(±)7(8)-epoxy-4Z,10Z,13Z,16Z,19Z-docosapentaenoic acid] | DHA | 4.09 | 0.2 | 400 |
| mws-oxlipid-N5 | 9,10-EpOME[(±)9,10-epoxy-12Z-octadecenoic acid] | LA | 3.95 | 0.2 | 400 |
| mws-oxlipid-N6 | 12,13-EpOME[(±)12(13)epoxy-9Z-octadecenoic acid] | LA | 3.89 | 0.2 | 400 |
| mws-oxlipid-N7 | 5-isoPGF2VI[(8β)-5,9α,11α-trihydroxy-prosta-6E,14Z-dien-1-oic acid] | ARA | 0.86 | 0.2 | 400 |
| mws-oxlipid-N8 | 9-HOTrE[9S-hydroxy-10E,12Z,15Z-octadecatrienoic acid] | ALA | 2.40 | 0.2 | 400 |
| mws-oxlipid-N9 | 13-HOTrE[13S-hydroxy-9Z,11E,15Z-octadecatrienoic acid] | ALA | 2.51 | 0.2 | 400 |

RT, retention time; LLOQ, Lower Limit of quantitation; ULOQ, Upper Limit of quantitation.

## Table S4. Clinical characteristics of patients with severe sepsis and health controls

| **Group** | **Control**  **N=16** | **Sepsis**  **N=16** | **P value** |
| --- | --- | --- | --- |
| Age, years | 57.5±9.7 | 54.6±12.3 | 0.468 |
| Male | 8 (50%) | 8 (50%) | - |
| White blood cell, 10^9^/L | 7.0±2.1 | 19.3±8.9 | <0.001 |
| Neutrophil, 10^9^/L | 4.6±2.0 | 17.3±8.1 | <0.001 |
| Neutrophil, % | 63.9±8.3 | 87.4±9.5 | <0.001 |
| Monocyte, 10^9^/L | 0.36±0.08 | 0.48±0.31 | 0.163 |
| Monocyte, % | 5.4±1.6 | 2.9±1.8 | <0.001 |
| Alanine aminotransferase, U/L | 29.2±13.1 | 78.9±65.0 | 0.005 |
| Aspertate aminotransferase, U/L | 17.4±7.3 | 219.3±303.3 | 0.012 |
| Urea nitrogen, mmol/L | 4.6±1.1 | 14.5±8.5 | <0.001 |
| Serum creatinine, μmol/L | 64.6±14.1 | 193.3±127.7 | <0.001 |
| LDH, U/L | 185.1±32.5 | 694.3±682.5 | 0.006 |
| CK-MB, μg/L | 0.52±0.35 | 12.8±26.4 | 0.073 |
| Melatonin, μg/L | 405.5±102.2 | 132.5±32.5 | <0.001 |

Variables are presented as number (proportion) or mean ± SD. P value was estimated with chi-square test or Student's t-test.

## Video S1-4. The video of the process of UTMD-mediated CMB blast

Representative ultrasound contrast images of CMBs in the heart before (Video S1) and after plasmid/CMBs injection (Video S2), ultrasound-targeted microbubble blast (Video S3) and after the process (Video S4).
